# Supplementary material for: NIPMAP: niche-phenotype mapping of multiplex histology data by community ecology
Source: Nat Commun. 2023 Nov 7;14:7182. doi: 10.1038/s41467-023-42878-z (PMC10630431; doi:10.1038/s41467-023-42878-z)
Supplement: Supplementary file 1 — Supplementary Information [file 41467_2023_42878_MOESM1_ESM.pdf]

Supplementary Information

Anissa El Marrahi, Fabio Lipreri, Ziqi Kang, Louise Gsell,
Alper Eroglu, David Alber, Jean Hausser

1 Supplementary figures

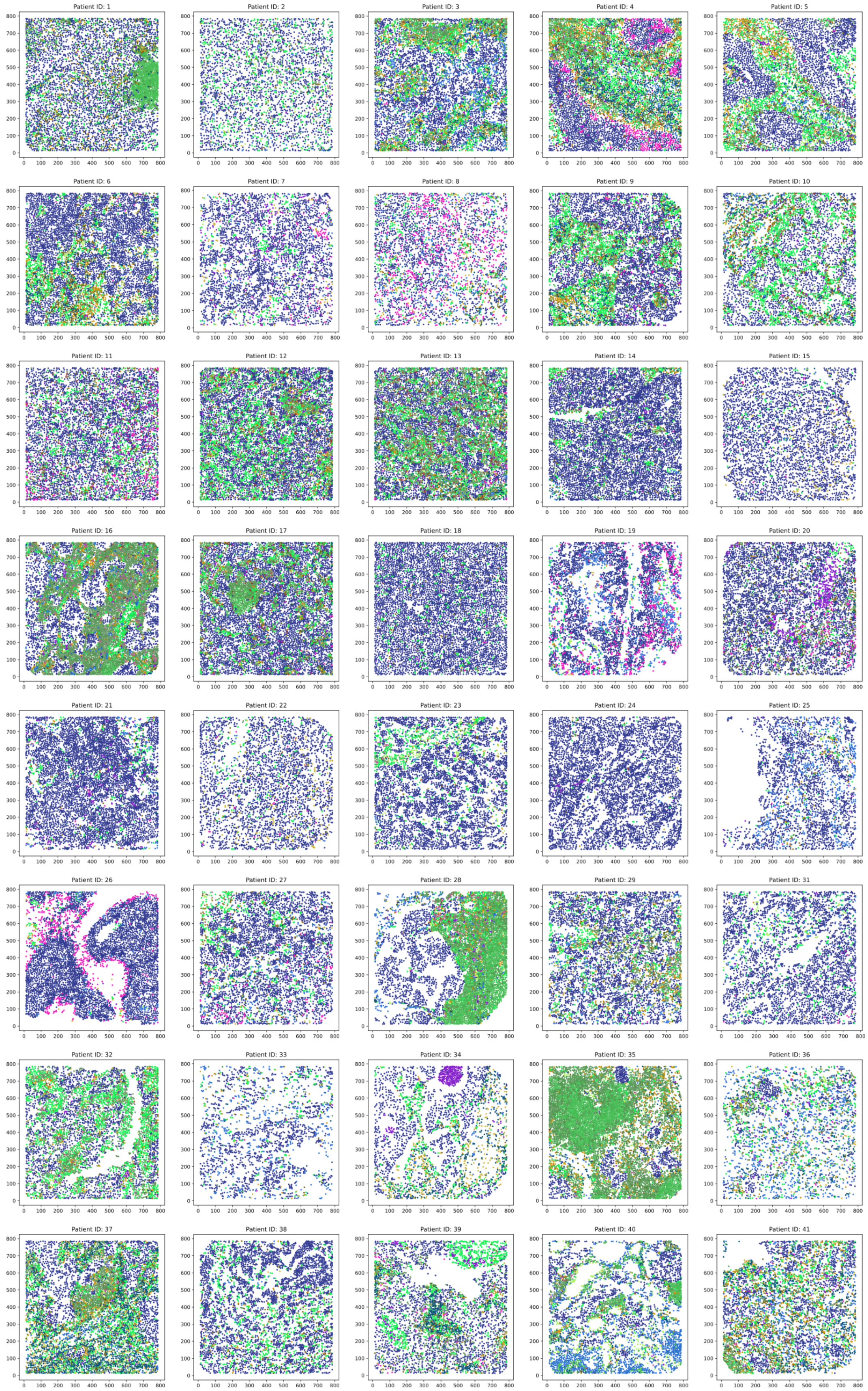

Supplementary Figure 1: Tumor architecture is difficult to interpret due to its many cell types and strong inter-sample variability. Each dot represents a cell and cells are colored according to type. Data: Keren et al. [1]

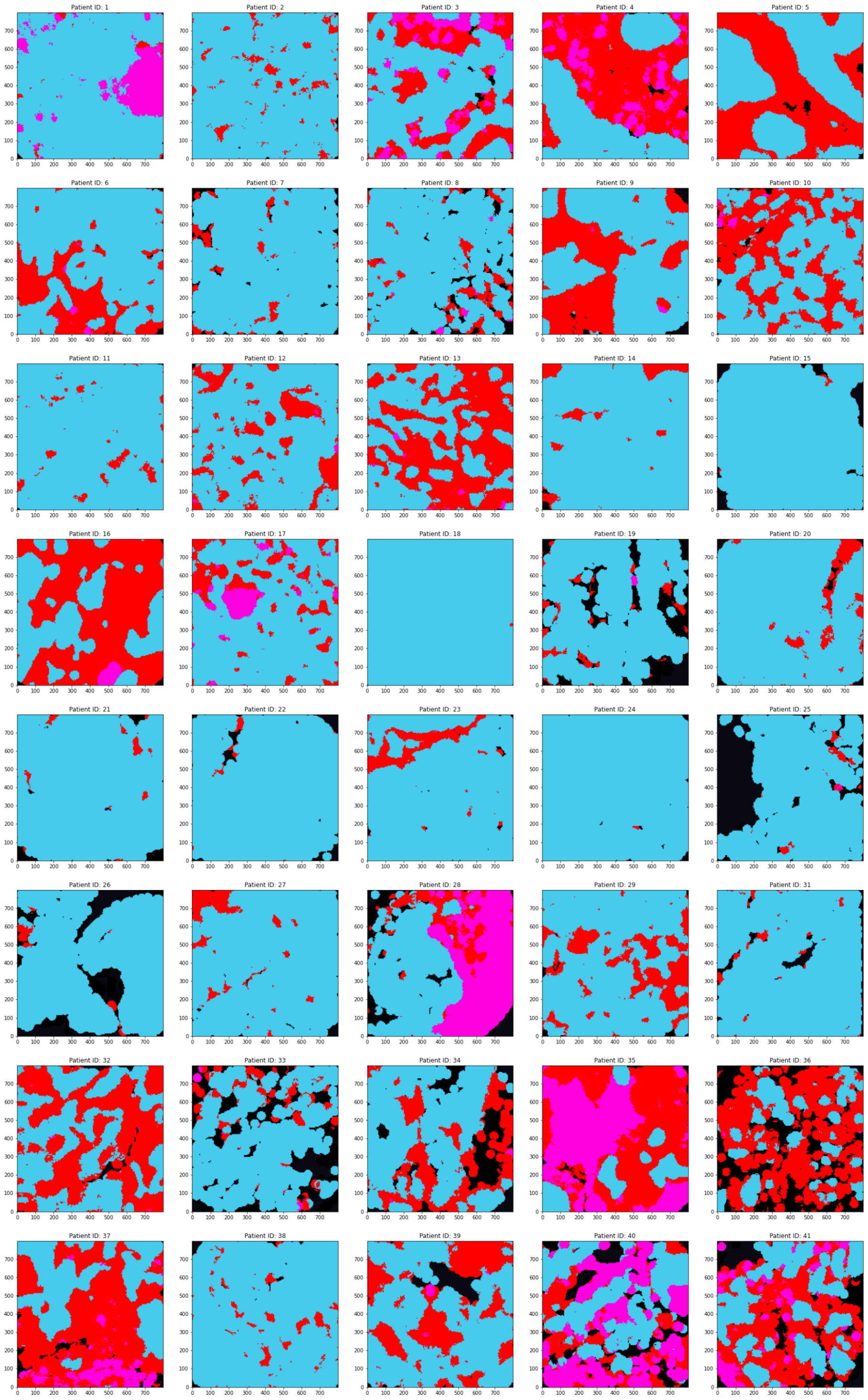

Supplementary Figure 2: A mix of four niches explain spatial variation in cellular abundance on 40 patients with TNBC. The 40 samples of Keren et al. [1] were annotated in terms of niches: blue is cancer, red is inflammatory, pink is TLS, black is fibrotic.

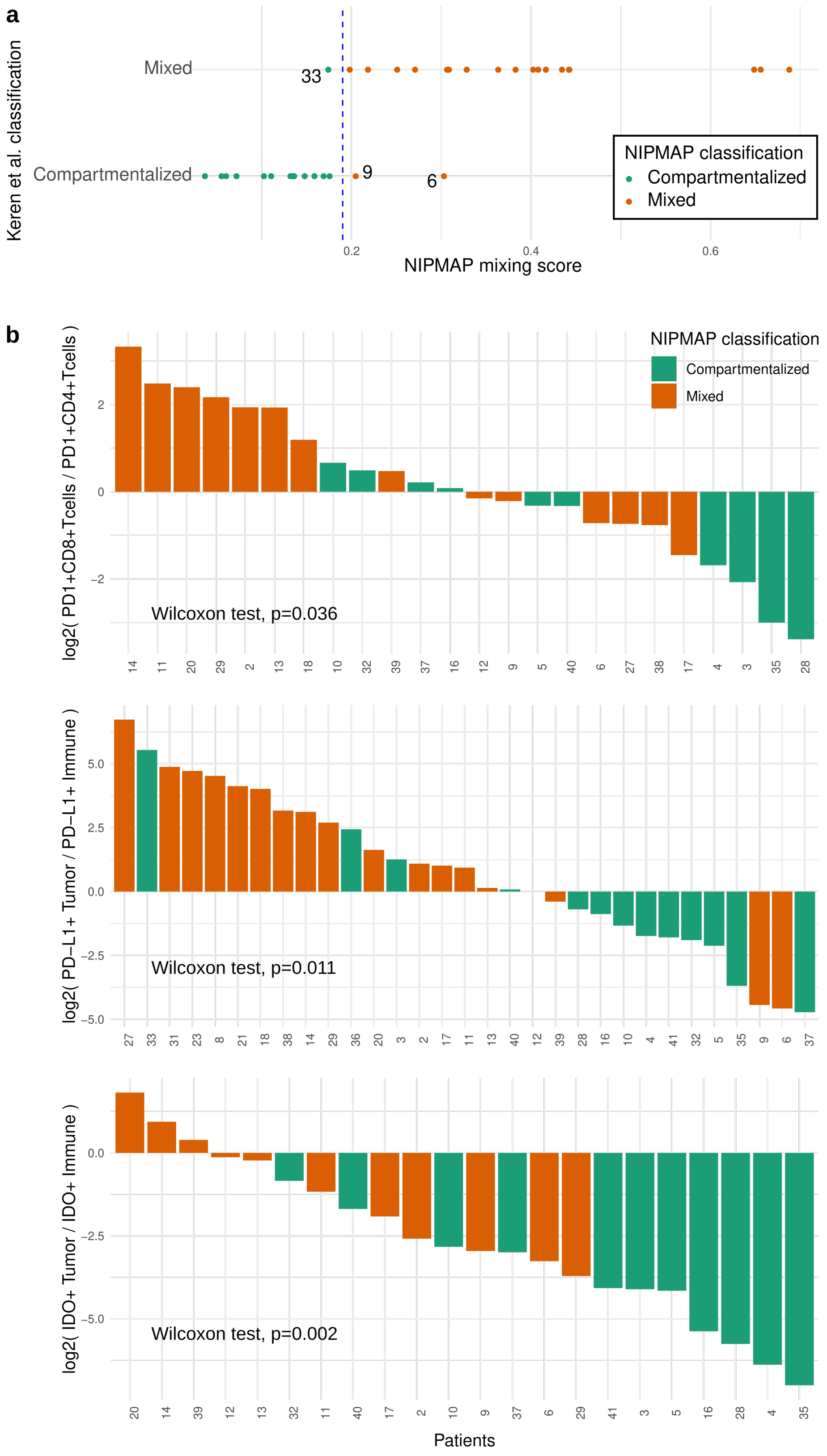

Supplementary Figure 3: NIPMAP niches capture the mixed vs compartimentalized breast tumor architectures uncovered by Keren et al. [1]. **a.** The NIPMAP mixing score (x-axis) reproduces the Compartmentalized-Mixed classification of Keren et al. [1] for 37 out of 40 samples. **b.** The NIPMAP mixing score reproduces associations between the Mixed-Compartmentalized classification of samples and the immuno-regulatory signaling environment previously reported by Keren et al. [1]. Wilcoxon/Mann-Whitney rank sum test with $n = 24, 31, 22$, bilateral.

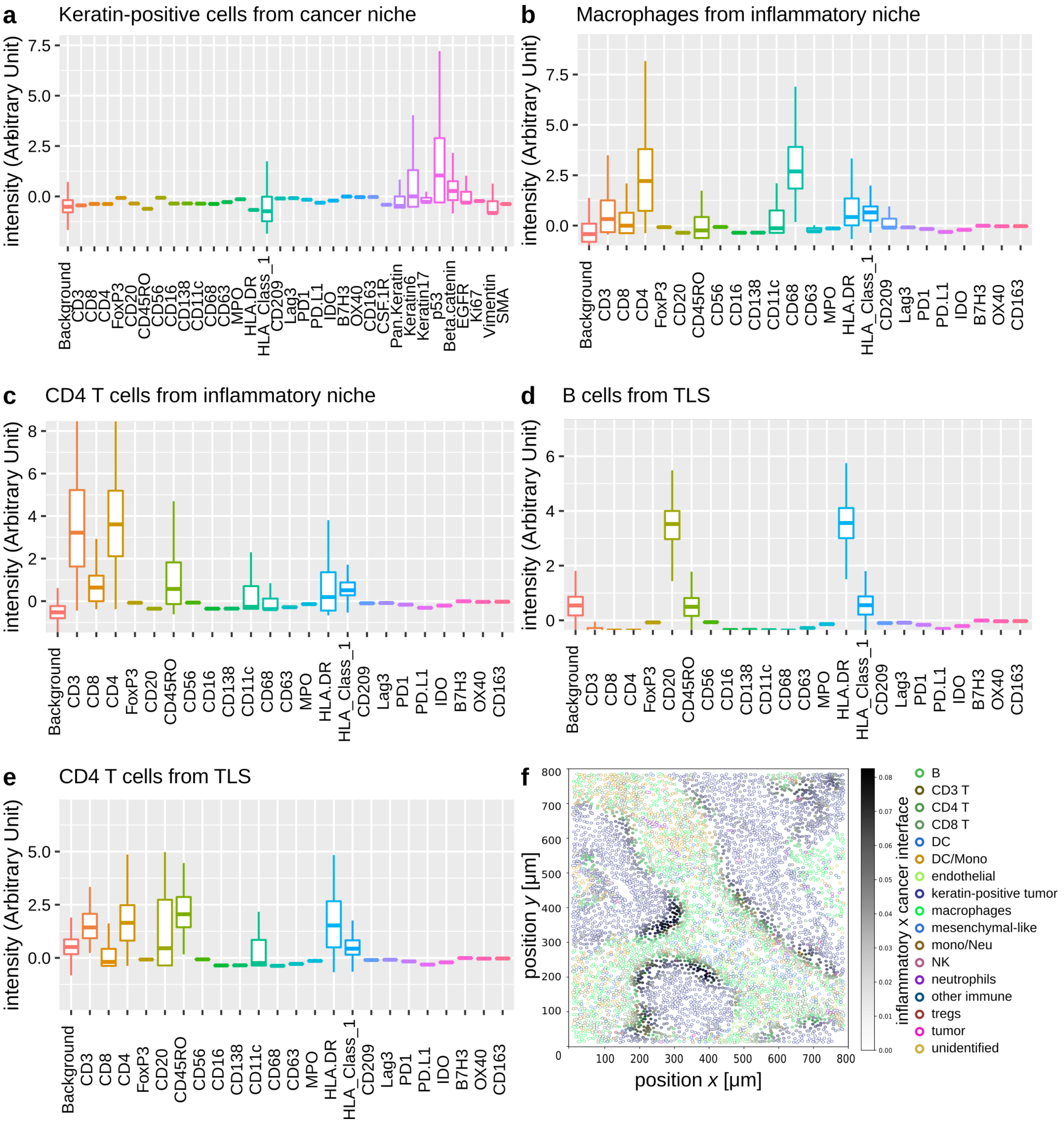

Supplementary Figure 4: Spatial phenotyping of different cell types in the data of Keren et al. [1]. **a-e.** Cells from the TME express different phenotypic markers depending on the niche in which they are found. **f.** Cells located at the interface between any two niches — here the cancer and inflammatory niches — are identified as cells where the weight of these two niches and thus their product is large. Note that the cancer-inflammatory interface is less visually compelling in this panel compared to Fig. 2f. For example, in particular in the central area at the top of the tissue, green (immune) cells border pink (cancer) cells, yet the area is not strongly marked as an interface. This is because cellular density is lower where green meets pink, suggesting that a fibrotic/necrotic area separates the cancer from the inflammatory niche. This local variability in cellular density can be corrected for (Methods) to identify interface regions more faithful to visual impression (Fig. 2f).

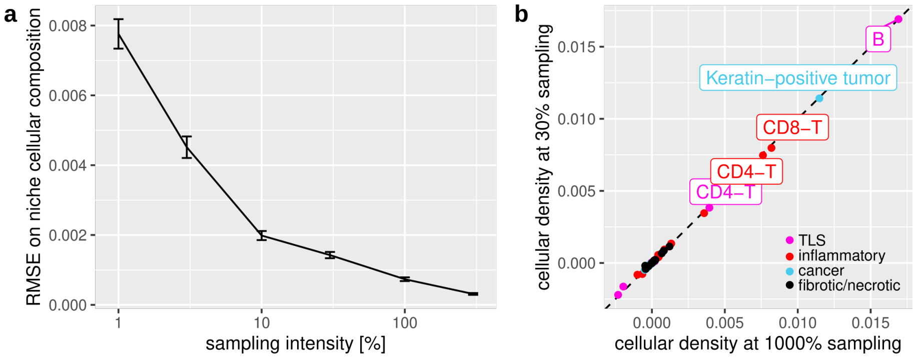

Supplementary Figure 5: Sampling only a fraction of the tissue can speed up computation at minimal loss of accuracy in niche composition. **a.** Increasing sampling intensity (ratio of the total site area over the tissue area) decreases the error on the cellular composition of niches. A sampling intensity of 10% or more is sufficient to control the error in this simulation. Error bars: standard error, $n = 100$ for each sampling intensity. **b.** A sampling intensity of 30% — as used in our re-analysis of the MIBI data of Keren et al. [1] — is sufficient to accurately determine the cellular composition of niches. The cellular composition of niches inferred by sampling at 30% and 1000% intensities correlate highly ($R > 0.99$). Dots represent different cell types in specific niches (color).

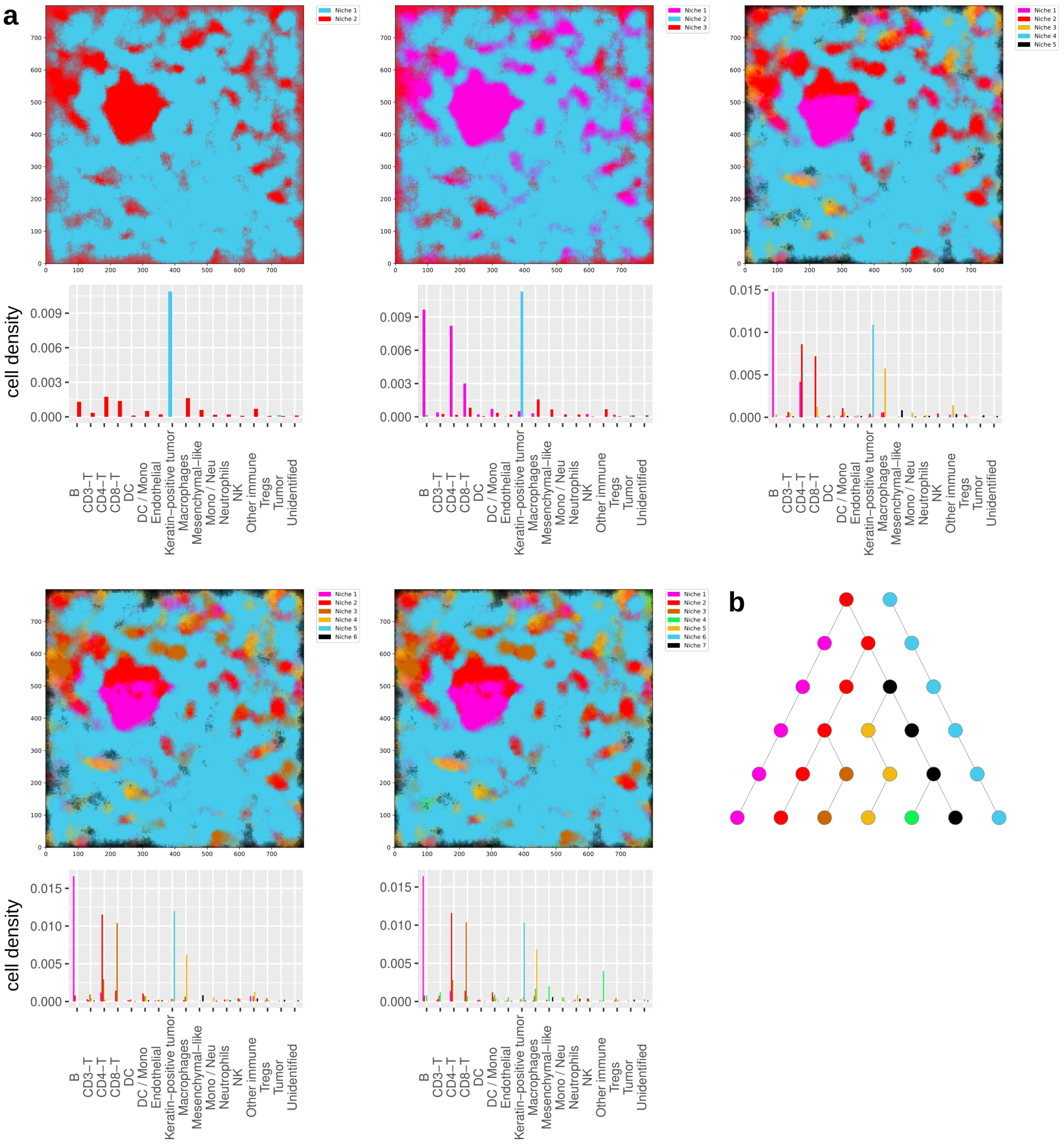

Supplementary Figure 6: Increasing the number of niches produces finer-grained niches. **a.** Tissue sections show the niche segmentation of Sample 17 using 2-7 niches. For 4 niches, see Supplementary Fig. 1b and Fig. 2g. A two-niche segmentation of the tissue finds a tumor and a stromal niche. Adding a niche stratifies the stromal niche into a B / T CD4 rich region (pink) and a stromal niche with lower cellular density (red). Adding a fourth niche splits the red niche into an inflammatory (red) and fibrotic/necrotic niche (black). Adding a fifth niche identifies a macrophage sub-niche (orange) within the fibrotic/necrotic (black) niche. A sixth niche splits the inflammatory niche (red) into a T CD4-rich (red) and a T CD8-rich niche (brown). Adding a seventh niche highlights a sub-niche rich in other immune cells (green) in the fibrotic/necrotic niche (black). **b.** A tree represents the pattern of successive niche splits as the number of niches grows.

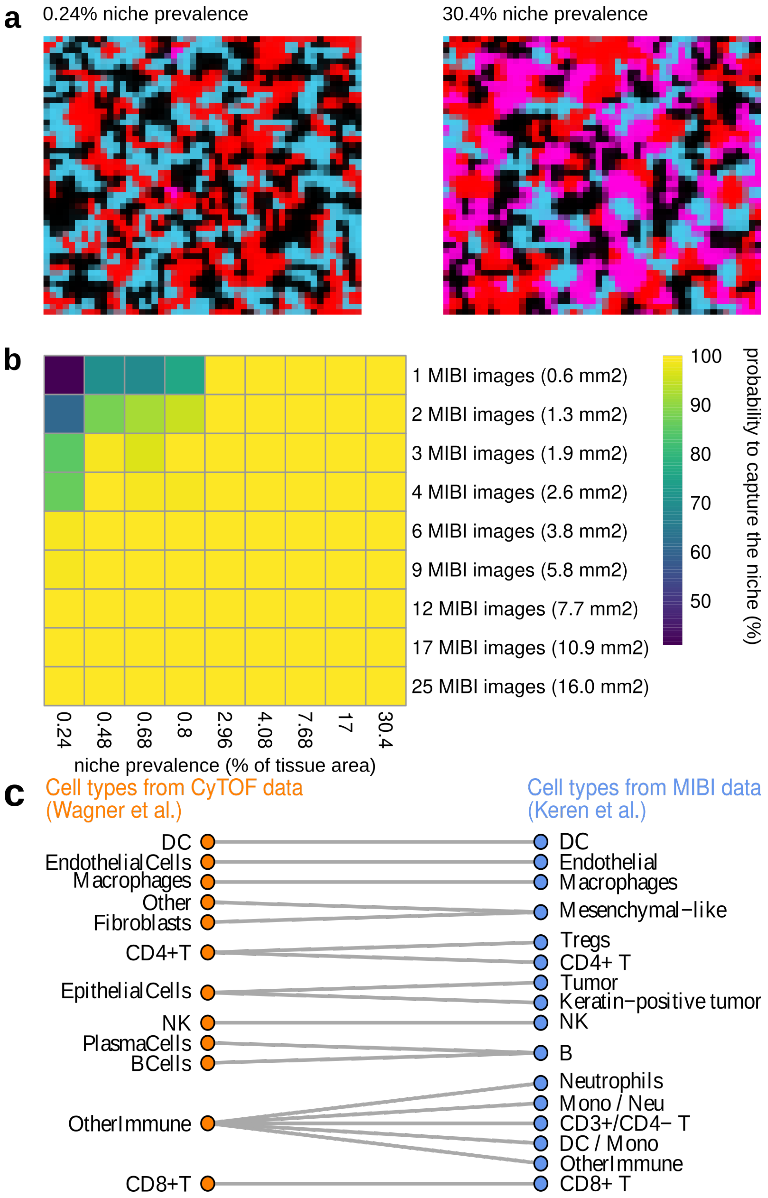

Supplementary Figure 7: NIPMAP can identify rare niches from limited tissue samples. **a.** The spatial distribution of four niches (colors) was simulated using a 4-species reaction-diffusion partial differential equations model. The initial condition was varied so as to simulate tissues in which one niche was more rare than the three other niches. **b.** The probability to capture a rare niche increases with niche prevalence (columns) and with the total tissue area available for niche-phenotype mapping (simulated MIBI images as rows). **c.** To compare the micro- and macro-architecture, cell types were mapped into a set of cell types common to MIBI (Keren et al., in blue) and CyTOF (Wagner et al., in orange) data.

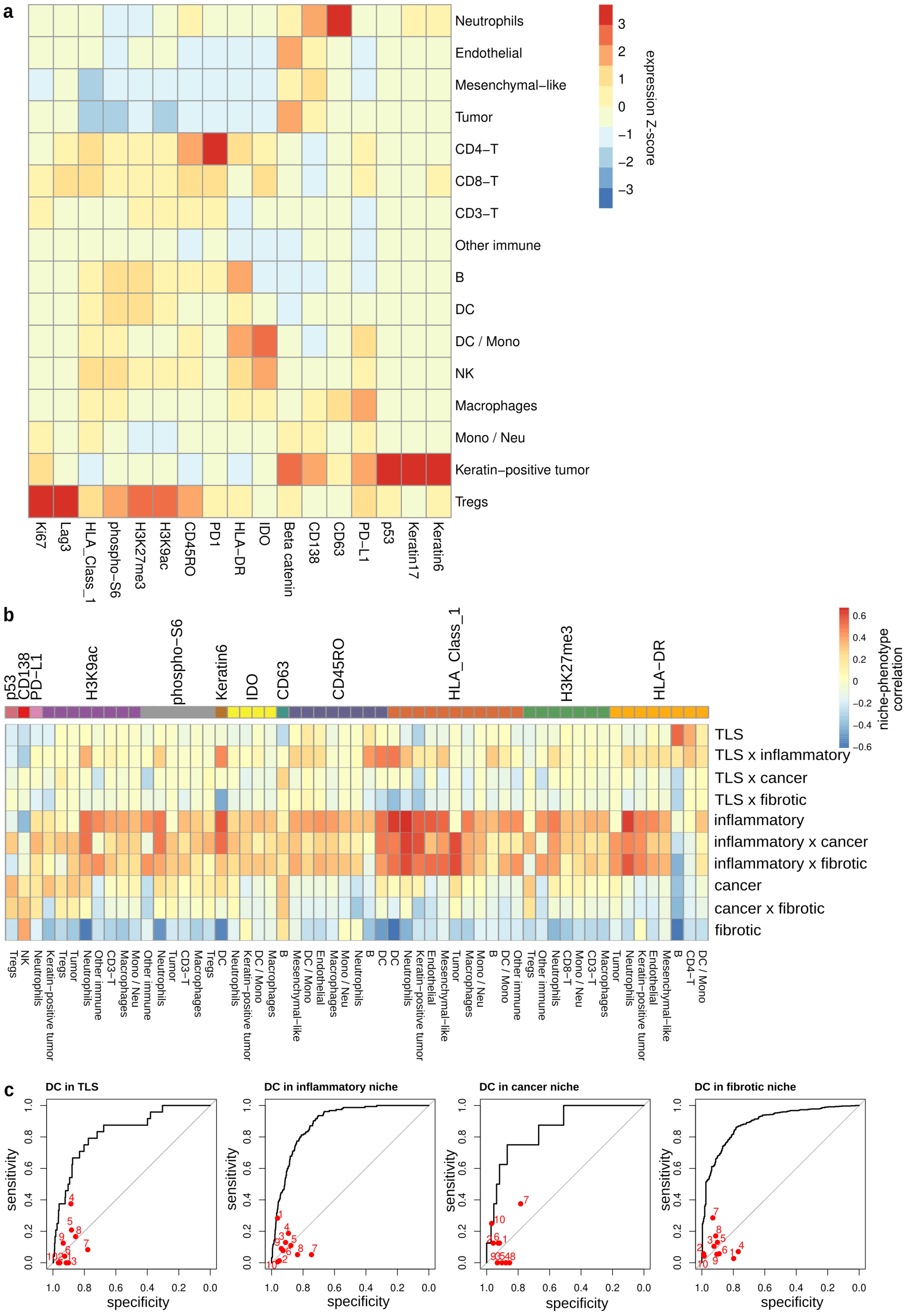

a
Neutrophils
Endothelial
Mesenchymal−like
Tumor
CD4−T
CD8−T
CD3−T
Other immune
B
DC
DC / Mono
NK
Macrophages
Mono / Neu
Keratin−positive tumor
Tregs
Ki67
Lag3
HLA_Class_1
phospho−S6
H3K27me3
H3K9ac
CD45RO
PD1
HLA−DR
IDO
Beta catenin
CD138
CD63
PD−L1
p53
Keratin17
Keratin6
expression Z-score
3
2
1
0
−1
−2
−3
b
p53
CD138
PD−L1
H3K9ac
phospho−S6
Keratin6
IDO
CD63
CD45RO
HLA_Class_1
H3K27me3
HLA−DR
niche-phenotype correlation
0.6
0.4
0.2
0
−0.2
−0.4
−0.6
TLS
TLS x inflammatory
TLS x cancer
TLS x fibrotic
inflammatory
inflammatory x cancer
inflammatory x fibrotic
cancer
cancer x fibrotic
fibrotic
Tregs
NK
Neutrophils
Keratin−positive tumor
Tregs
Tumor
Neutrophils
Other immune
CD3−T
Macrophages
Mono / Neu
Other immune
Neutrophils
Tumor
CD3−T
Macrophages
Tregs
DC
Neutrophils
Keratin−positive tumor
DC / Mono
Macrophages
B
Mesenchymal−like
DC / Mono
Endothelial
Macrophages
Mono / Neu
Neutrophils
B
DC
DC
Neutrophils
Keratin−positive tumor
Endothelial
Mesenchymal−like
Tumor
Macrophages
Mono / Neu
B
DC / Mono
Other immune
Tregs
Other immune
Neutrophils
CD8−T
Mono / Neu
CD3−T
Tumor
Macrophages
Neutrophils
Keratin−positive tumor
Endothelial
Mesenchymal−like
B
CD4−T
DC / Mono
c
DC in TLS
DC in inflammatory niche
DC in cancer niche
DC in fibrotic niche
sensitivity
specificity
0.0
0.2
0.4
0.6
0.8
1.0

d

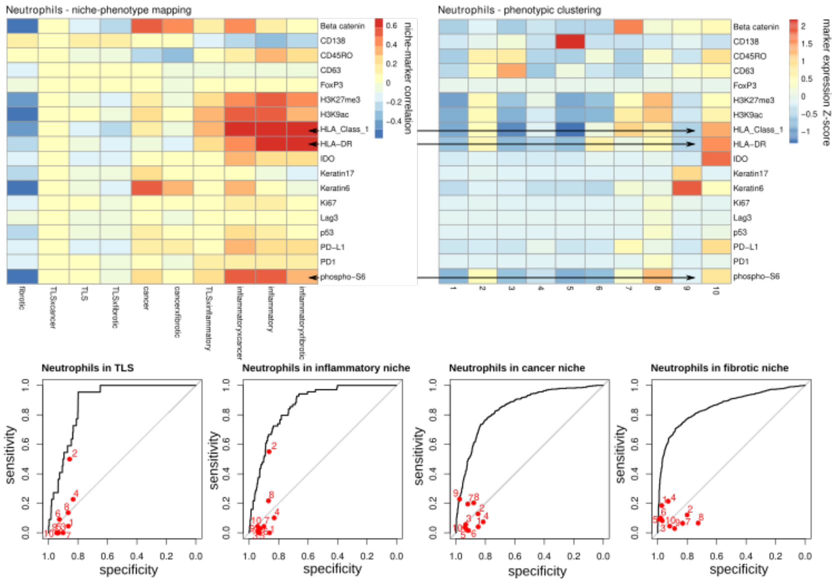

e

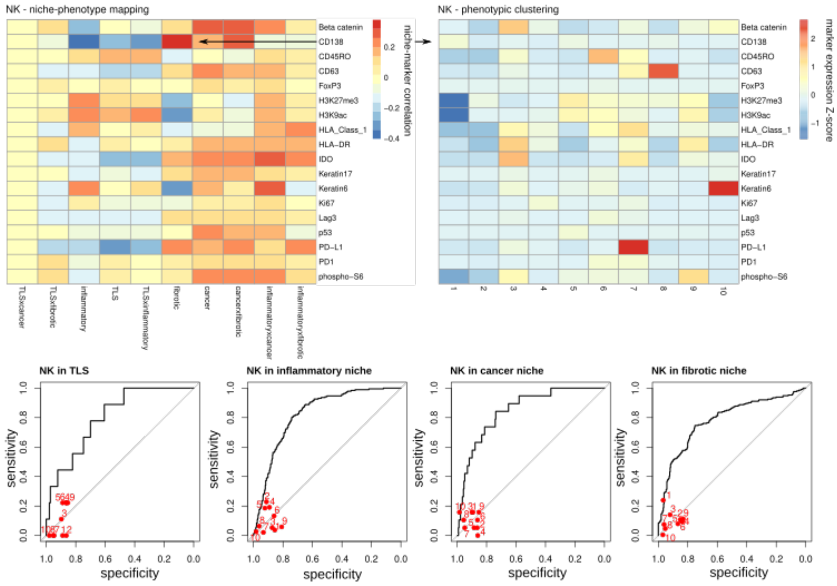

Supplementary Figure 8: a. Keren et al. profiled 18 phenotypic marker to characterize the function of cells in their local environment. The heatmap summarizes the expression patterns of phenotypic markers across the different cell types of the dataset after Z-scoring and hierarchical bi-clustering. **b.** NIPMAP finds associations between cell phenotypes and niches/interfaces illustrated by a heatmap organized by phenotypic markers. **c.** Predictions of niche from phenotypic markers based on a linear model (black curves) and phenotypic clustering (red dots) for DCs in all four niches. In each niche, a phenotypic cluster predicts cell location as precisely as a linear model of niche location from all markers. This supports the hypothesis that the spatial context of cells is a stronger determinant of phenotype than cell-autonomous effects: if cell-autonomous factors dominated phenotypic heterogeneity, (spatially agnostic) phenotypic clusters would associate poorly with space. **d-e.** Observations in neutrophils and NK cells are consistent with the hypothesis that the spatial context of cells is a stronger determinant of phenotype than cell-autonomous effects: if cell-autonomous factors dominated phenotypic heterogeneity, (spatially agnostic) phenotypic clusters would associate poorly with space. As with dendritic cells (see Fig. 4), (a) markers highlighted by phenotypic clustering overlap but differ from markers identified by niche-phenotype mapping, (b) some spatial markers found by niche-phenotype mapping are missed by phenotypic clustering, (c) in each niche, a phenotypic cluster predicts niche location as accurately as niche phenotype mapping, though with low sensitivity. **d.** Top: in neutrophils, niche-phenotype mapping (left) and (spatially-agnostic) phenotypic clusters (right) identify common phenotypic markers — HLA-I, HLA-DR, phospho-S6. Some spatial markers are not found by phenotypic clustering (CD45RO). Some makers identified by phenotypic clustering don't associate with space (CD138). Below: predictions of niche from phenotypic markers based on a linear model (black curves) and phenotypic clustering (red dots). **e.** Top: in NK cells, niche-phenotype mapping (left) and (spatially-agnostic) phenotypic clusters (right) identify common phenotypic markers — CD63, IDO, PD-L1. Some spatial markers are not found by phenotypic clustering (CD138). Below: predictions of niche from phenotypic markers based on a linear model (black curves) and phenotypic clustering (red dots).

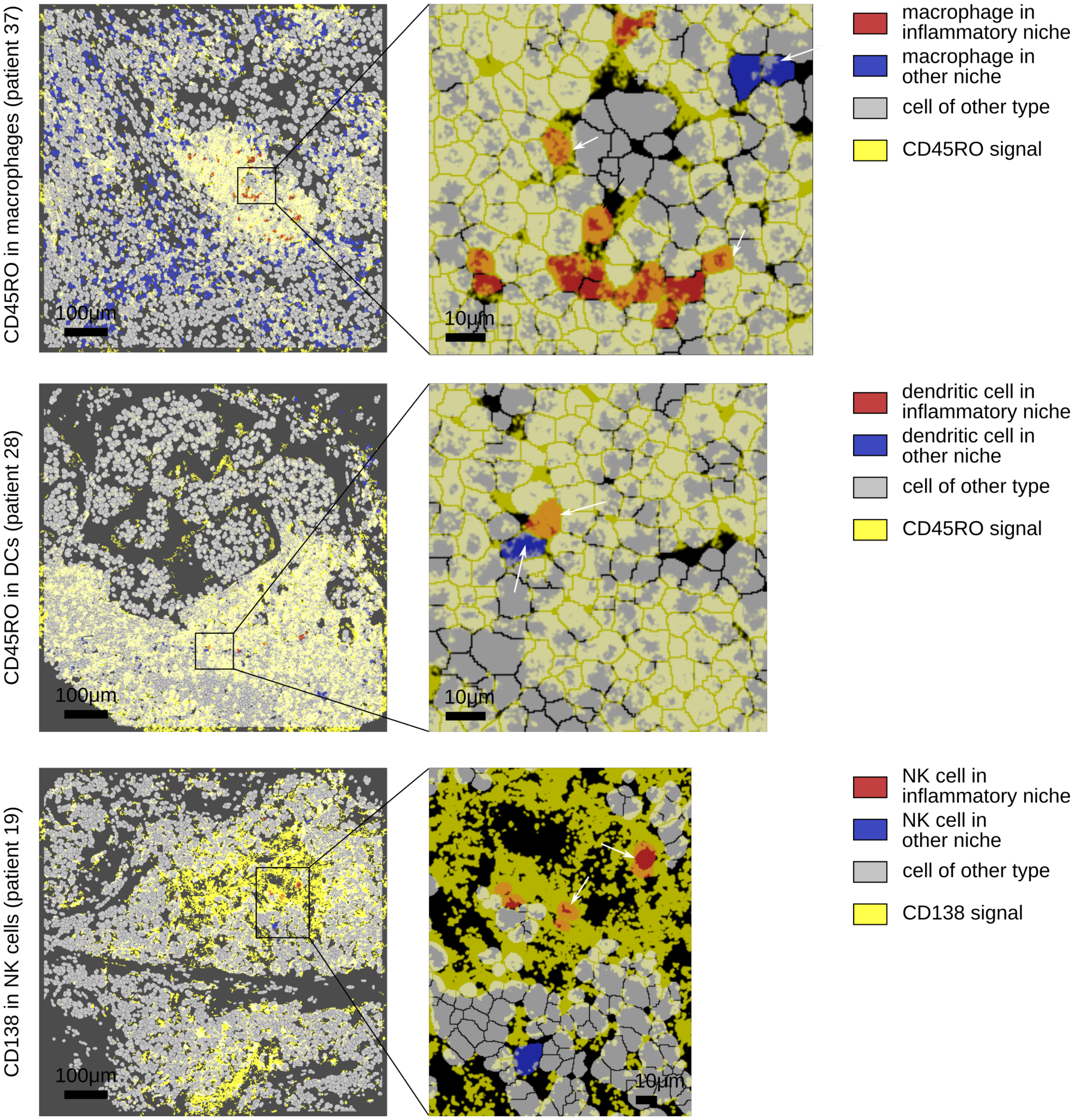

Supplementary Figure 9: Overlaying marker signal and niche-specific cellular segmentation masks to validate spatial phenotypes suggested by niche-phenotype mapping. **Row 1** Visual inspection supports CD45RO expression by macrophages localized in the inflammatory niche (red). CD45RO signal localizes in membrane regions of macrophages, including in macrophages that contact neighboring cells with less or no CD45RO signal. The CD45RO signal in macrophages cannot be explained by spatial spill-over because marker signal is expected to spill from cells with higher marker abundance over to neighboring cells. That spatial signal spill-over is unlikely is confirmed by macrophages outside the inflammatory niche (blue): these macrophages have less CD45RO signal as expected, despite being in contact with a CD45RO+ cell. **Row 2** Illustration of CD45RO expression by DCs localized in the inflammatory niche. Shown is a dendritic cell (red) with more systematic CD45RO signal than neighboring cells. The CD45RO signal in that cell cannot be explained by spatial spill-over because marker signal is expected to spill from cells with higher marker abundance over to neighboring cells. That spatial signal spill-over is unlikely is confirmed by a DCs outside the inflammatory niche (blue): this cell has less CD45RO signal as expected, despite being in contact with a CD45RO+ cell. **Row 3** Visual inspection suggests that the CD138 signal in NK cells of the fibrotic niche is explained by a tendency of NK cells to localize in CD138-rich areas of the fibrotic niche. CD138, also known as Syndecan-1, is a membrane protein that binds growth factors, adhesion receptors, soluble small molecules, proteinases, and other ECM proteins [2]. The CD138 signal localizes in NK cells of the fibrotic niche with little contact with other cells, thus ruling out spatial spill-over. Most of CD138 signal appears to be extracellular. This observation is consistent with previous reports of CD138 expression in breast and other other solid tumors, mainly by fibroblasts and tumors cells [3, 4], and that the extracellular domain of CD138 can be proteolytically cleaved and released into the extracellular compartment to regulate inflammation and fibrosis [2].

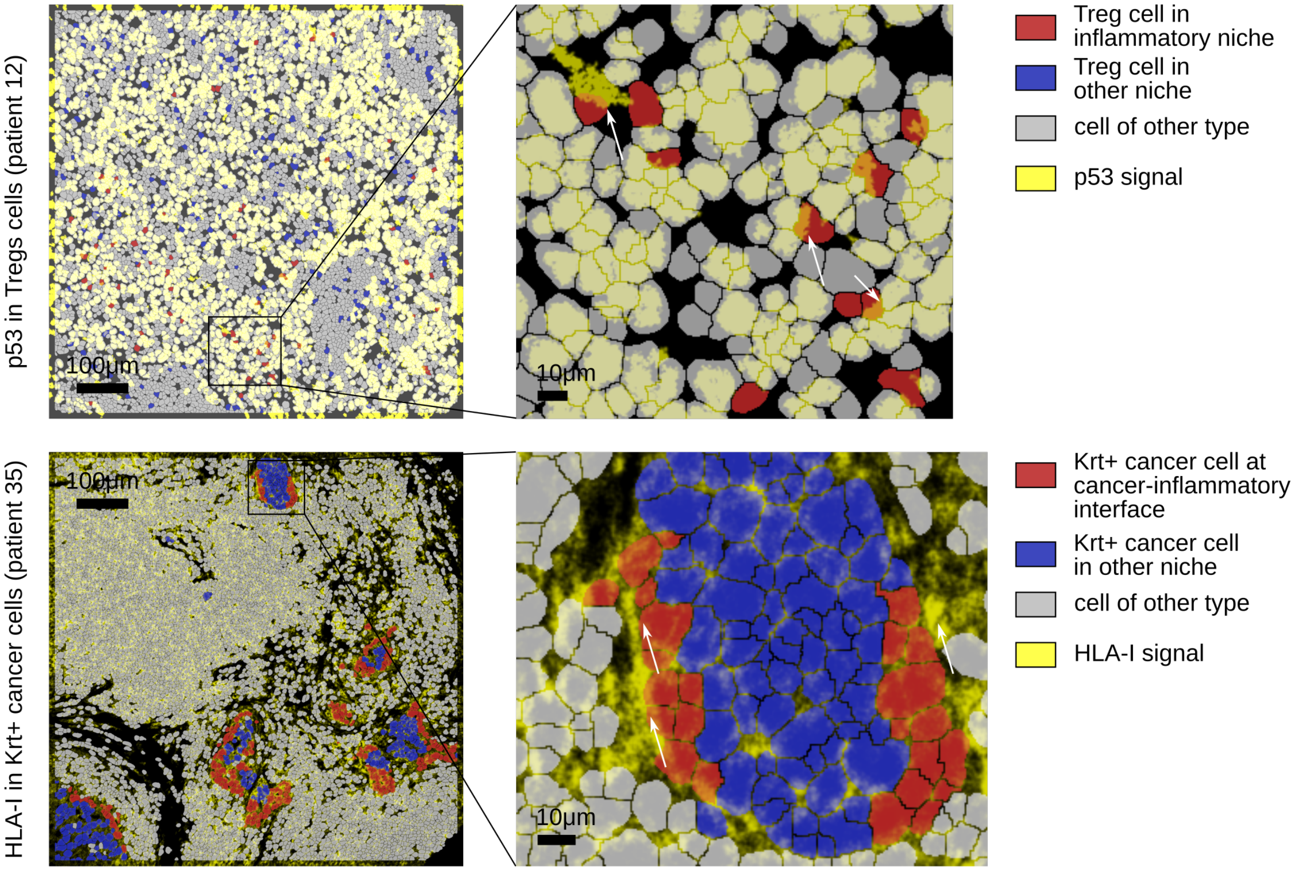

Supplementary Figure 10: Row 1. Visual inspection suggests that the p53 signal in Tregs of the cancer niche is likely due to spatial spill-over. Spatial spill-over is consistent with the localization of Tregs with partial p53 signal next to (cancer) cells with system p53 signal. A Treg is also observed in an area with apparent extra-cellular p53 signal. **Row 2.** Visual inspection suggests two hypotheses for the HLA-I signal in cancer cells at the cancer-inflammatory interface. One hypothesis is that cancer cells at the cancer-inflammatory interface express HLA-I. An alternative hypothesis is that HLA-I signal in that region is of extra-cellular origin, imputable to the soluble form of HLA-I [5].

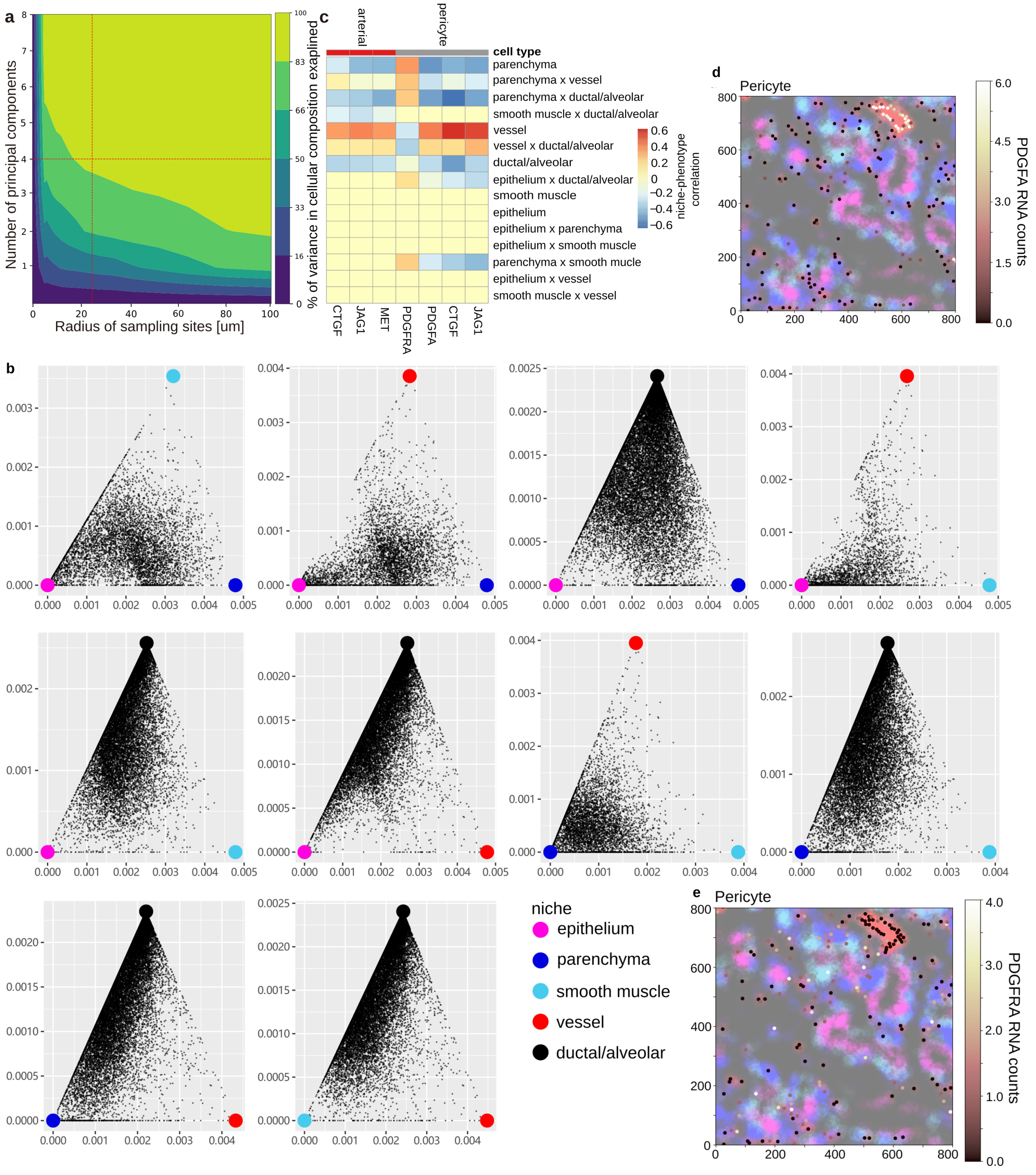

Supplementary Figure 11: **a.** In the data of Sountoulidis et al. [6], PCA explains 85% of the variance in the cellular composition of sites 25μm in radius projected on the first 4 principal components. **b.** Projections of sites onto planes defined by 3 out of the 5 niches allow visualization of data structure for dimensions greater than 3. **c.** Statistically significant niche-phenotype associations ($\rho > 0.3$ and $q < 1\%$). **d.** Pericytes express PDGFA when located in the vessel niche but not when located in other niches. Dots: pericytes. Color bar: PDGFA RNA count per cell. Background color: niche segmentation. **E.** Pericytes express PDGFRA when located in the parenchyma.

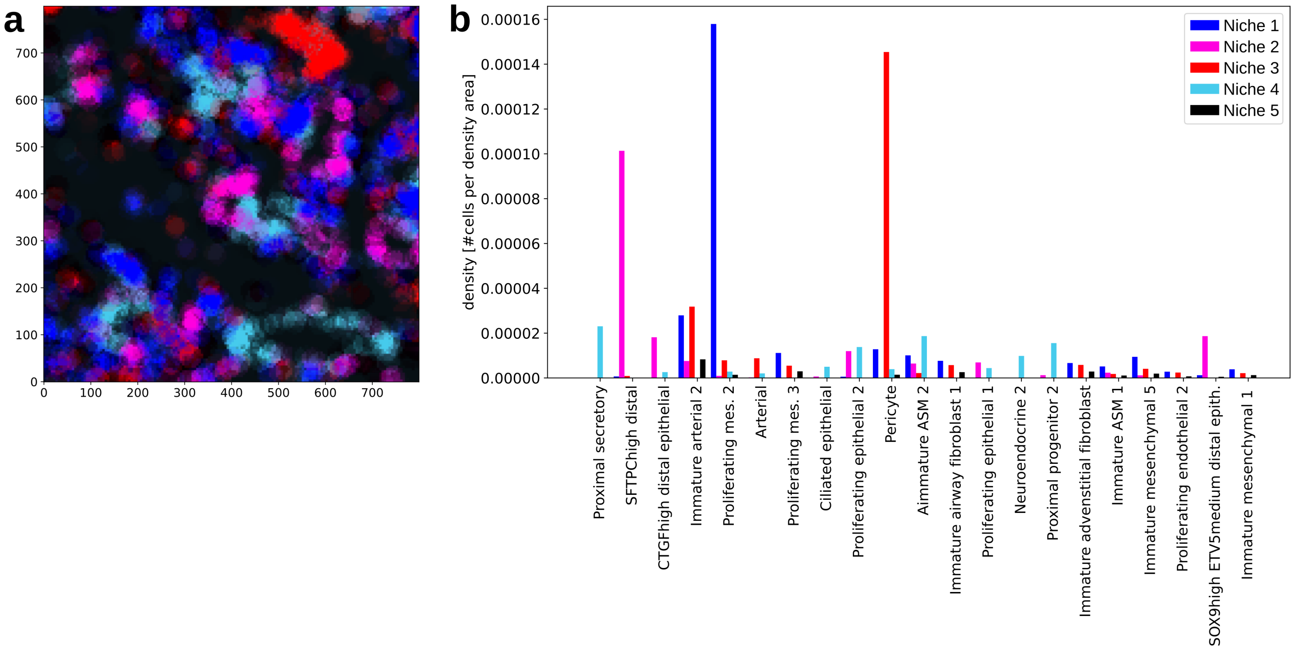

Supplementary Figure 12: **a.** Performing niche identification of the same tissue sample using 73 cell types and phenotypes produces the same tissue segmentation as with 32 cell types (compare to main Fig. 5b-c). **b.** Niche identification with 73 cell types identifies niches of similar cellular composition to 32 cell types: (1) an epithelial niche is dominated by epithelial cells, (2) a parenchymal niche characterized by a high prevalence of mesenchymal cells, (3) a smooth muscle niche populated by alveolar smooth muscle and secretory cells, (4) a vascular niche with pericytes and arterial cells, and (5) ductal and alveolar (liquid-filled) space.

Supplementary Note 1: Patient-dependent usage of the same niches explains inter-patient variation in the cellular composition of tumors

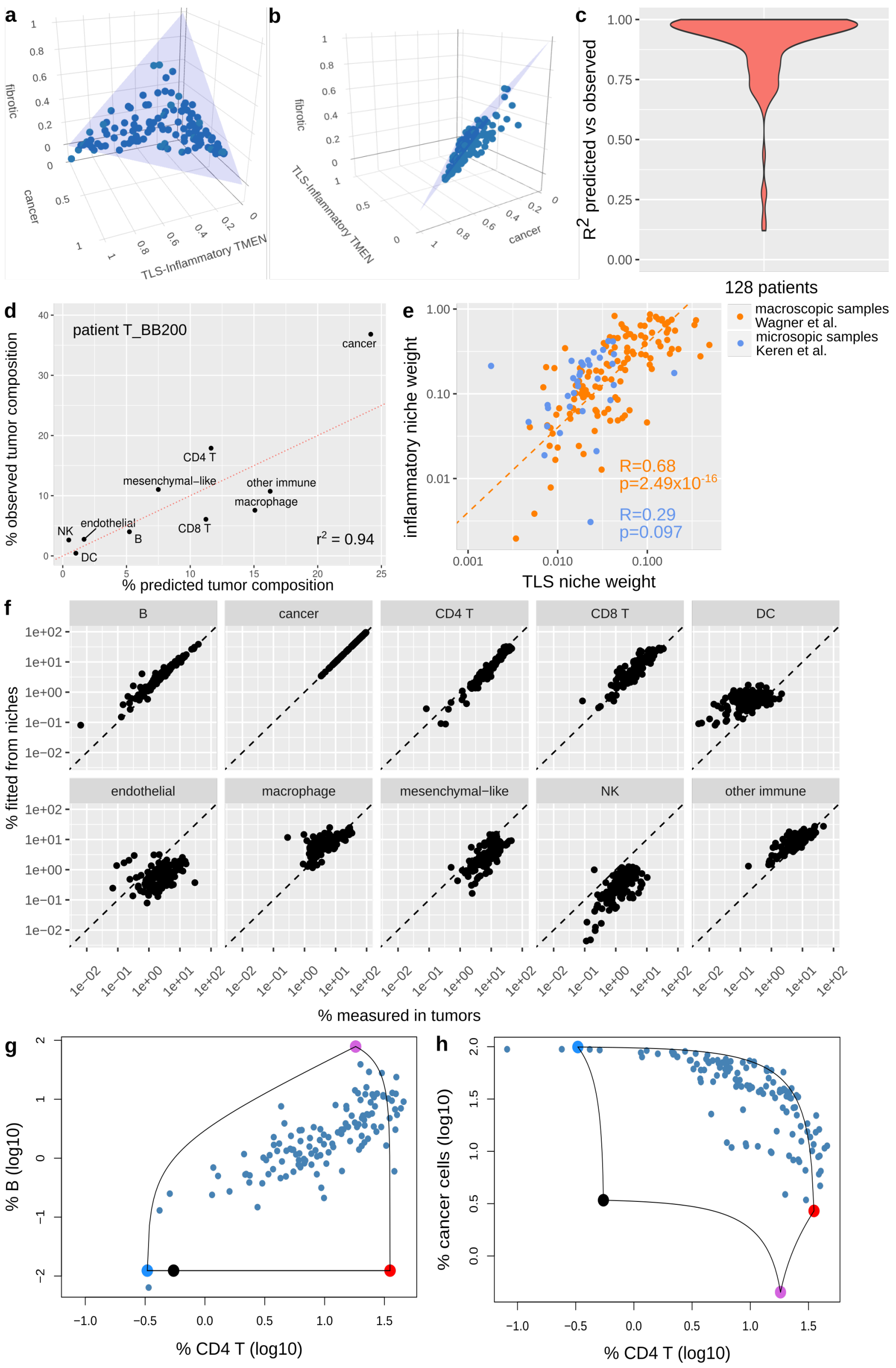

Supplementary Figure 13: Microscopic tumor niches explain inter-patient variation in the macroscopic cellular composition of breast tumors. **a-b**. Upon regressing tumor composition on the niches, the niches weights fall on a unit triangle, consistent with weights summing up to 1. This is expected if niches explain inter-patient variation in tumor macro-composition. **c-d**. Tumor macro-composition reconstructed from niches highly correlates with experimentally observed macro-composition (median patient in panel **d**). **e**. The inflammatory and TLS niches are coupled at the macro-scale but not at the micro-scale. Axes represent the weight of the inflammatory and TLS niches in a regression analysis of tumor sample composition (dots) on the inflammatory and TLS niches. Tumor samples were either macroscopic (orange, Wagner et al. [7]) or microscopic (blue, Keren et al. [1]). P-values from t test on Pearson's product moment correlation coefficient, $n = 40$ (Keren) or 128 samples (Wagner). **f**. Niches capture inter-patient variation in the macroscopic composition of tumors at both low and high cell type abundance. **g-h**. Inter-patient variation in the pairwise composition of cancer, CD4 and B cells is bounded by the niches, over two orders of magnitude.

If patient-dependent usage of the same niches explains inter-patient variation in the cellular composition of tumors, one expects that (a) regressing tumor composition on the niches produces weights that are positive and sum up to 1, and (b) tumor composition can be reconstructed based on the niches.

We find that this is the case. Regression weights fall on a triangular area that represents positive weights and a constraint of summing up to 1 (Fig. 13a-b). Tumor composition is reconstructed from the niches with low prediction error ($r^2 = 0.95$ on average across patients, Fig. 13c-d), including for cell types of low abundance (Fig. 13f). Thus, niches accurately model inter-tumor variation in macro-composition.

Methods for test (a): computing niche weights from macroscopic cellular abundance of tumors. To test if inter-patient variation in the macroscopic cellular composition of tumors can be modeled by patient-specific variation in the prevalence of universal cellular niches, we compute the contribution of the different niches to each macroscopic sample by regression analysis.

Let $\boldsymbol{C}$ be the vector of cellular composition of one tumor and $B = \{\boldsymbol{b_1}, \boldsymbol{b_2}, \boldsymbol{b_3}, \boldsymbol{b_4}\}$ a matrix of cells proportions for the four niches, with niches as column-vectors $\boldsymbol{b_i}$. If the cell composition of tumors $\boldsymbol{C}$ is constrained by a mix of niches, it can written as

$$\boldsymbol{C} = \sum_{i=1}^{4} \theta_i \boldsymbol{b_i} + \boldsymbol{\epsilon} \tag{1}$$

where θ_i represents the proportion of each niche in the tumor, $0 \leq \theta_i \leq 1$. $\boldsymbol{\epsilon}$ represents the error.

With a dataset of many tumors, we can write this equation in a matrix form:

$$C = \Theta B^T. \tag{2}$$

Here, B has dimensions of cell types x niches. Θ, with dimensions of samples x niches, is the unknown in this equation. We solve for Θ using the pseudo-inverse method. To do so, we first multiply both sides by B^T to obtain

$$B^T C^T = B^T B \Theta^T. \tag{3}$$

And we solve for Θ,

$$\Theta = ((B^T B)^{-1}(CB)^T)^T. \tag{4}$$

Since $(B^T B)$ is a square matrix, we can find its inverse if $det(B^T B) \neq 0$. We numerically find the inverse of $B^T B$ using the `solve()` function in data analysis software R.

Methods for test (b): testing that tumor composition can be reconstructed based on the niches We assess the accuracy of the linear regression by computing the coefficient of determination $\boldsymbol{R}$ for each sample,

$$\boldsymbol{R} = cor(C, \Theta B^T). \tag{5}$$

R_i represents the correlation between the cell proportions observed and predicted from a linear combination of niches in patient i.

Supplementary Note 2: There is macroscopic coupling between the prevalence of the TLS and inflammatory niches in tumors

The observation that the TLS and inflammatory niches occupy the same corner of the simplex could be explained if their prevalence is coupled in tumors. To test this hypothesis, we regress inter-patient variation in the macroscopic cellular composition of tumors on the TLS and inflammatory niches. Regression analysis indeed reveals a positive correlation between the weights of the two niches (Fig. 13e, $r = 0.68$, $p = 2.49 \times 10^{-16}$, $n = 128$ samples, t test on Pearson's product moment correlation coefficient). This suggests a macroscopic coupling between the TLS and inflammatory niches, with a ratio of 3.5 area units of inflammatory niche for each area unit of TLS niche. This coupling does not have its origin in microscopic constraints: performing the same analysis on the cellular composition of the 40 microscopic samples of Keren et al. [1], we do not observe a coupling between the TLS and inflammatory niches (Fig. 13e, $r = 0.29$, $p = 0.097$, $n = 40$ samples, t test on Pearson's product moment correlation coefficient).

Supplementary Note 3: Niches constrain the cellular composition of tumors and capture dependencies in tumor macro-composition

Niches can be used to explain why certain combinations of cells are found in tumors and why others can never be observed. We find that the structure of cellular composition is strongly constrained by tumor micro-architecture. For example, while cellular abundance spans two orders of magnitude in variation, none of the 128 breast tumors of Wagner et al. [7] have high cancer cell content and high CD4 T cell content (Fig. 13h). This is because cancer cells and CD4 T belong to different niches and thus their co-occurrence is limited by the areas occupied by these niches. Similarly, there cannot be tumors with many B cells but no CD4 cells: these two cell types occupy the same TLS niche, thus their abundance has to be coupled (Fig. 13g).

The observation that niches identified from microscopic tumor architecture explain inter-patient variation in cellular composition further supports their relevance in interpreting tissue architecture: it implies specific rules of tumor architecture and many rules of tumor architecture are incompatible with our observations. For example, if different tumors employ different niches — analogous to genomics, where different tumors have different mutations — microscopic cellular composition does not fall on a single low-dimensional simplex but on more complex shapes. Microscopic tumor cellular composition is not expected to fall on a low-dimensional simplex either if the cellular architecture of tumors is homogeneous, without spatial structure. If the prevalence of a given niche is constant across tumors, microscopic niches are not expected to tightly bound the scatter of macroscopic tumor samples in the visualization of Fig. 2k but instead land at a distance. Thus, our observations are consistent with the view that breast tumors are built using four niches, shared across patients, and whose prevalence are variable and sample-specific.

Supplementary Note 4: niches can potentially be identified based on spatial marker intensities data without segmenting and without assigning predefined types to cells

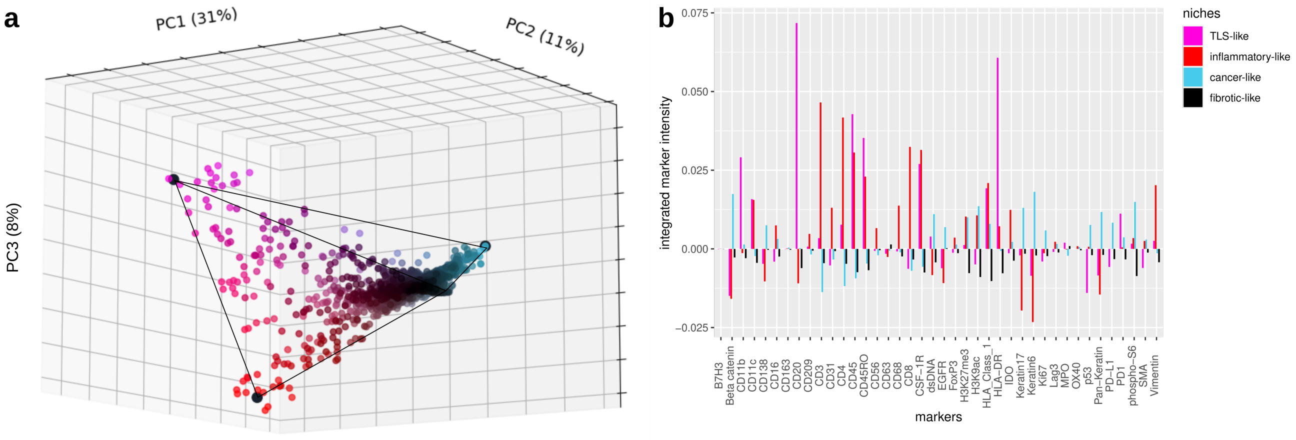

Supplementary Figure 14: The niche architecture of tissues constrains local integrated marker intensities to a simplex, potentially allowing automatic niche identification from cell-free marker data using the same methods as cell-based niche identification. **a.** Projecting the marker intensities integrated up over cells from 4000 sampling sites of the MIBI data of Keren et al. [1] on their 3 principal components suggests the geometry of a simplex. **b.** Fitting the simplex of integrated marker intensities (Q matrix, see text) suggests that its endpoints represent the same niches as the niches identified from cell-type-based niche identification.

NIPMAP requires assigning a type to each cell based on its marker intensity profiles and prior knowledge, with potential downsides in terms of biasing niches according to prior knowledge of cell types as well as time and efforts in segmenting individual cells and assigning types to them.

To address this, one can ground niche identification in marker intensities of local tissue regions prior to segmenting and assigning types to individual cells. Doing so is expected to be feasible if each (potentially unknown) cell type expresses a specific set of markers in a given (potentially unknown) niche.

To see why, we note that cell-type-based niche identification rests on the hypothesis that spatial heterogeneity in a tissue arises because the local weight of niches $\boldsymbol{\alpha}$ varies in space, so that cellular composition $\boldsymbol{x}$ at a given site can be written as a weighted average of the cellular composition of the different niches B,

$$\boldsymbol{x} = B\boldsymbol{\alpha}, \tag{6}$$

with α_i the local weight of niche i and $\sum_i \alpha_i = 1$. Here B is a matrix of dimensions cell types x niches with the cellular composition of each niche.

Collecting the intensity of markers expressed by cells of a given type into a matrix M of dimensions markers x cell types, we can write the intensities $\boldsymbol{y}$ of the different markers integrated over all cells at a given site as

$$\boldsymbol{y} = MB\boldsymbol{\alpha} = Q\boldsymbol{\alpha}, \tag{7}$$

where $Q := MB$ represents the integrated marker intensities of each niche. Thus, integrated marker intensities at multiple sites are expected to fall on a low-dimensional simplex in the space of marker intensities, the marker equivalent of the cellular composition simplex of Fig. 2. Multiplying the cellular composition simplex by the matrix M of cell-type-specific marker intensities transforms it into the marker intensity simplex. The endpoints of the marker intensity simplex represent the tissue niches.

The implication is that performing archetype analysis on the integrated marker intensities at many sites should uncover tissue niches. Note that we do *not* need to know the number of cell types, the type of a given cell, nor the markers expressed by each cell type to estimate the marker composition of the different niches Q by archetype analysis. Since the analysis is done on marker intensities integrated over the cells present at the site, segmenting the tissue into cells is potentially optional.

We tested this idea using the MIBI data of Keren et al. [1]. We used the same methodology as in the analyses of Fig. 2 except for one point: instead of counting the number of cells of each type weighted by a Gaussian kernel ($\sigma = 25\mu$m), we summed up the intensities of each of the 34 markers over the cells present at a given site, weighted by the Gaussian kernel.

Projecting the sites on 3 PCs shows that sites arrange themselves as a simplex (Fig. 14a), consistent with expectations. Inferring the endpoints of the simplex by archetype analysis identifies four niches whose markers are reminiscent of the niches identified by analyzing cellular composition (Fig. 14b, Fig. 2g). One niche is characterized by high expression of CD20, a B cell marker, suggesting a TLS. The second niche features high expression of CD68, a macrophage marker, and of CD3, CD8 and CD4, suggesting an inflammatory niche. The third niche expresses different Keratin markers, suggesting a cancer niche. The fourth niche shows low expression of all markers and could thus represent the fibrotic/necrotic niche.

These observations suggest that niche identification can potentially be performed without assigning predetermined types to cells and in unsegmented tissue samples.

Supplementary references

[1] Leeat Keren, Marc Bosse, Diana Marquez, Roshan Angoshtari, Samir Jain, Sushama Varma, Soo-Ryum Yang, Allison Kurian, David Van Valen, Robert West, Sean C. Bendall, and Michael Angelo. A Structured Tumor-Immune Microenvironment in Triple Negative Breast Cancer Revealed by Multiplexed Ion Beam Imaging. *Cell*, 174(6):1373–1387.e19, September 2018. ISSN 00928674. doi: 10.1016/j.cell.2018.08.039. URL `https://linkinghub.elsevier.com/retrieve/pii/S0092867418311000`. Publisher: Elsevier.

[2] Sandeep Gopal. Syndecans in Inflammation at a Glance. *Frontiers in Immunology*, 11:227, February 2020. ISSN 1664-3224. doi: 10.3389/fimmu.2020.00227. URL `https://www.ncbi.nlm.nih.gov/pmc/articles/PMC7040480/`.

[3] Simon Kind, Christina Merenkow, Franziska Büscheck, Katharina Möller, David Dum, Viktoria Chirico, Andreas M. Luebke, Doris Höflmayer, Andrea Hinsch, Frank Jacobsen, Cosima Göbel, Sören Weidemann, Christoph Fraune, Christina Möller-Koop, Claudia Hube-Magg, Till S. Clauditz, Ronald Simon, Guido

Sauter, Waldemar Wilczak, Ahmed Abdulwahab Bawahab, Jakob R. Izbicki, Daniel Perez, and Andreas Marx. Prevalence of Syndecan-1 (CD138) Expression in Different Kinds of Human Tumors and Normal Tissues. *Disease Markers*, 2019:4928315, December 2019. ISSN 0278-0240. doi: 10.1155/2019/4928315. URL `https://www.ncbi.nlm.nih.gov/pmc/articles/PMC6954471/`.

[4] Palaiologou, Marina, Delladetsima, Ioanna, and Tiniakos, Dina. CD138 (syndecan-1) expression in health and disease. *Histology and Histopathology*, (29):177–189, January 2014. ISSN 0213-3911. doi: 10.14670/HH-29.177. URL `https://doi.org/10.14670/HH-29.177`.

[5] Amy L. Kessler, Marco J. Bruno, and Sonja I. Buschow. The Potential of Soluble Human Leukocyte Antigen Molecules for Early Cancer Detection and Therapeutic Vaccine Design. *Vaccines*, 8(4):775, December 2020. ISSN 2076-393X. doi: 10.3390/vaccines8040775. URL `https://www.ncbi.nlm.nih.gov/pmc/articles/PMC7766713/`.

[6] Alexandros Sountoulidis, Sergio Marco Salas, Emelie Braun, Christophe Avenel, Joseph Bergenstråhle, Jonas Theelke, Marco Vicari, Paulo Czarnewski, Andreas Liontos, Xesus Abalo, Žaneta Andrusivová, Reza Mirzazadeh, Michaela Asp, Xiaofei Li, Lijuan Hu, Sanem Sariyar, Anna Martinez Casals, Burcu Ayoglu, Alexandra Firsova, Jakob Michaëlsson, Emma Lundberg, Carolina Wählby, Erik Sundström, Sten Linnarsson, Joakim Lundeberg, Mats Nilsson, and Christos Samakovlis. A topographic atlas defines developmental origins of cell heterogeneity in the human embryonic lung. *Nature Cell Biology*, January 2023. ISSN 1465-7392, 1476-4679. doi: 10.1038/s41556-022-01064-x. URL `https://www.nature.com/articles/s41556-022-01064-x`.

[7] Johanna Wagner, Maria Anna Rapsomaniki, Stéphane Chevrier, Tobias Anzeneder, Claus Langwieder, August Dykgers, Martin Rees, Annette Ramaswamy, Simone Muenst, Savas Deniz Soysal, Andrea Jacobs, Jonas Windhager, Karina Silina, Maries van den Broek, Konstantin Johannes Dedes, Maria Rodríguez Martínez, Walter Paul Weber, and Bernd Bodenmiller. A Single-Cell Atlas of the Tumor and Immune Ecosystem of Human Breast Cancer. *Cell*, 177(5):1330–1345.e18, May 2019. ISSN 1097-4172. doi: 10.1016/j.cell.2019.03.005. URL `http://www.ncbi.nlm.nih.gov/pubmed/30982598`. Publisher: Elsevier.
