# Supplementary material for: NIPMAP: niche-phenotype mapping of multiplex histology data by community ecology
Source: Nat Commun. 2023 Nov 7;14:7182. doi: 10.1038/s41467-023-42878-z (PMC10630431; doi:10.1038/s41467-023-42878-z)
Supplement: Supplementary file 3 — Description of Additional Supplementary Files [file 41467_2023_42878_MOESM3_ESM.pdf]

Description of Additional Supplementary Files

Supplementary Data 1
Description: Table mapping the fine-grained cell types of Sountoulidis et al. to the simplified cell types used in the present analysis.
