# Supplementary material for: NIPMAP: niche-phenotype mapping of multiplex histology data by community ecology
Source: Nat Commun. 2023 Nov 7;14:7182. doi: 10.1038/s41467-023-42878-z (PMC10630431; doi:10.1038/s41467-023-42878-z)
Supplement: Supplementary file 5 — Reporting Summary [file 41467_2023_42878_MOESM5_ESM.pdf]

nature portfolio

Corresponding author(s): Jean Hausser

Last updated by author(s): Sep 20, 2023

Reporting Summary

Nature Portfolio wishes to improve the reproducibility of the work that we publish. This form provides structure for consistency and transparency in reporting. For further information on Nature Portfolio policies, see our Editorial Policies and the Editorial Policy Checklist.

Statistics

For all statistical analyses, confirm that the following items are present in the figure legend, table legend, main text, or Methods section.

n/a	Confirmed	
☐	☒	The exact sample size (*n*) for each experimental group/condition, given as a discrete number and unit of measurement
☐	☒	A statement on whether measurements were taken from distinct samples or whether the same sample was measured repeatedly
☐	☒	The statistical test(s) used AND whether they are one- or two-sided<br>*Only common tests should be described solely by name; describe more complex techniques in the Methods section.*
☐	☒	A description of all covariates tested
☐	☒	A description of any assumptions or corrections, such as tests of normality and adjustment for multiple comparisons
☐	☒	A full description of the statistical parameters including central tendency (e.g. means) or other basic estimates (e.g. regression coefficient) AND variation (e.g. standard deviation) or associated estimates of uncertainty (e.g. confidence intervals)
☐	☒	For null hypothesis testing, the test statistic (e.g. *F*, *t*, *r*) with confidence intervals, effect sizes, degrees of freedom and *P* value noted<br>*Give P values as exact values whenever suitable.*
☒	☐	For Bayesian analysis, information on the choice of priors and Markov chain Monte Carlo settings
☒	☐	For hierarchical and complex designs, identification of the appropriate level for tests and full reporting of outcomes
☐	☒	Estimates of effect sizes (e.g. Cohen's *d*, Pearson's *r*), indicating how they were calculated

Our web collection on statistics for biologists contains articles on many of the points above.

Software and code

Policy information about availability of computer code

Data collection	No software was used for data collection: the present work is based on analysis of existing data.
Data analysis	All analyses were performed using R 4.3.1 and Python 3.7.13.<br>R packages include: tidyverse 2.0.0, ggplot2 3.4.3, ade4 1.7, factoextra 1.0.7, plotly 4.10.2, igraph, 1.5, reshape2 1.4.4, ggrepel 0.9.3, viridis 0.6.3, fdrtool 1.2.17, pheatmap 1.0.12, cluster 2.1.4, broom 1.0.5, pROC 1.18.4, devtools 2.4.5, ggrid 0.5.4<br>Python packages include: matplotlib 3.7.1, scipy 1.10.1, pandas 1.3.5, numpy 1.8.1, scikit-learn 0.0.post5, seaborn 0.12.2, qpsolvers 1.9.<br>All data necessary to reproduce the analysis have been uploaded to the github repository https://www.github.com/jhausserlab/NIPMAP

For manuscripts utilizing custom algorithms or software that are central to the research but not yet described in published literature, software must be made available to editors and reviewers. We strongly encourage code deposition in a community repository (e.g. GitHub). See the Nature Portfolio guidelines for submitting code & software for further information.

Data

Policy information about availability of data

All manuscripts must include a data availability statement. This statement should provide the following information, where applicable:

- Accession codes, unique identifiers, or web links for publicly available datasets
- A description of any restrictions on data availability
- For clinical datasets or third party data, please ensure that the statement adheres to our policy

The data to reproduce the analyses can be downloaded at https://github.com/jhausserlab/NIPMAP
Source data are provided with this paper.

Research involving human participants, their data, or biological material

Policy information about studies with human participants or human data. See also policy information about sex, gender (identity/presentation), and sexual orientation and race, ethnicity and racism.

Reporting on sex and gender	Most of the data (Wagner Cell 2019, Keren Cell 2018) we reanalyze in the present paper concern breast cancer, a disease with a prevalence 100x higher in females compared to males. The methodological principles developped in the article are expected to apply to males because, in both males and females (as well as multi-cellular organisms in general), cells self-organize into histological niches with functional specialization.
Reporting on race, ethnicity, or other socially relevant groupings	Not relevant: the experimental unit of the present study is a cell or groups of cells in a tissue, not human individuals. The study does not aim at investigating the characteristics of human participants but rather how to summarize the cellular and phenotypic architecture of tissues from multiplex imaging data.
Population characteristics	Keren et al. data: 41 samples, age 26-91 (mean 54.2 years); no gender information in cohort- presumably all female (99% of breast tumors patients are female)<br>Wagner et al. data: 145 patients, aged 29-93 (mean 62.8 years); 144 female, 1 male
Recruitment	The present study did not recruit participants but re-analyzed data acquired and published in previous studies. The original studies describe recruitment.
Ethics oversight	Ethical approval was obtained by the authors of the original studies that acquired and published the data when appropriate.

Note that full information on the approval of the study protocol must also be provided in the manuscript.

Field-specific reporting

Please select the one below that is the best fit for your research. If you are not sure, read the appropriate sections before making your selection.

[x] Life sciences [ ] Behavioural & social sciences [ ] Ecological, evolutionary & environmental sciences

For a reference copy of the document with all sections, see nature.com/documents/nr-reporting-summary-flat.pdf

Life sciences study design

All studies must disclose on these points even when the disclosure is negative.

Sample size	Not relevant: the present study does not aim at testing the statistical significance of a specific intervention nor associations between tissue features and patient outcomes but rather present new methodological principles to (a) summarize existing multiplex histology data and (b) highlight salient spatial features. We illustrate these principles on public data (n=40 breast tumors profiled by MIBI, n=1 fetal lung profiled by ISS, 128 tumors profiled by CyTOF). These datasets are sufficient to support our conclusion that NIPMAP suports interpreting multiplex histology data because they cover diverse technologies used in multiplex technologies (protein by MIBI/CyTOF vs RNA profiling by ISS) as well as health vs disease (fetal lung, cancer). Furthermore, two studies (Keren and Wagner) were chosen because they examine the same disease using different technologies, cohorts and were performed by different labs, thus allowing to verify the robustness of the NIPMAP methodology.
Data exclusions	Of the 41 breast tumor samples from Keren et al., patient 30 was excluded from our analysis following previous procedure by the authors (Keren et al., Cell 2018). Of the 144 samples of Wagner et al., one was excluded due to low quality (<50% of live cells) and 15 samples were excluded because they consisted mainly of non-cancer tissue and were thus inappropriate for use in the comparison to the tumor samples of Keren et al..
Replication	Results were reproduced by re-analyzing the data to confirm robustness of the identifed niches and niche-phenotype associations to the random positioning of sampling sites over the tissue and the number of sites used in the analysis. Robustness of the method was also assessed by analyzing two datasets from different organs (lung vs breast), different panel, different imaging technologies (protein vs RNA) and health vs disease. Finally, we evaluated the replicability of the findings with respect to changing the number of niches and the granularity of cell types used to identify these niches. We did not perform independent replicated experiments.
Randomization	Not relevant: the purpose of randomization is to prevent uncontrolled factors from biasing outcomes when testing whether a specific

	intervention causes a specific outcome. The present study does test whether specific interventions causes specific outcomes. Instead, it aims at introducing a new method to explore, interpret and summarize multiplex histology data.
Blinding	Not relevant. The purpose of blinding is to eliminate experimental biases that arise from a participants' expectations, observer's effect on the participants, observer bias or confirmation bias. However, here, we do not perform experiments. Instead, we introduce a computational method where participants are cells characterized by molecular markers which do not have the cognitive ability to have expectations or to be affected by the expectations of the analyst since the data was collected by independent experimentalists, years before the analyst first saw them. As it stands, our method is immune to observer and confirmation bias because it reports statistical significant patterns independently of any sample groups.

Reporting for specific materials, systems and methods

We require information from authors about some types of materials, experimental systems and methods used in many studies. Here, indicate whether each material, system or method listed is relevant to your study. If you are not sure if a list item applies to your research, read the appropriate section before selecting a response.

Materials & experimental systems

n/a	Involved in the study
☒	☐ Antibodies
☒	☐ Eukaryotic cell lines
☒	☐ Palaeontology and archaeology
☒	☐ Animals and other organisms
☒	☐ Clinical data
☒	☐ Dual use research of concern
☒	☐ Plants

Methods

n/a	Involved in the study
☒	☐ ChIP-seq
☒	☐ Flow cytometry
☒	☐ MRI-based neuroimaging
