# Supplementary figures and images for: NIPMAP: niche-phenotype mapping of multiplex histology data by community ecology
Source: Nat Commun. 2023 Nov 7;14:7182. doi: 10.1038/s41467-023-42878-z (PMC10630431; doi:10.1038/s41467-023-42878-z)

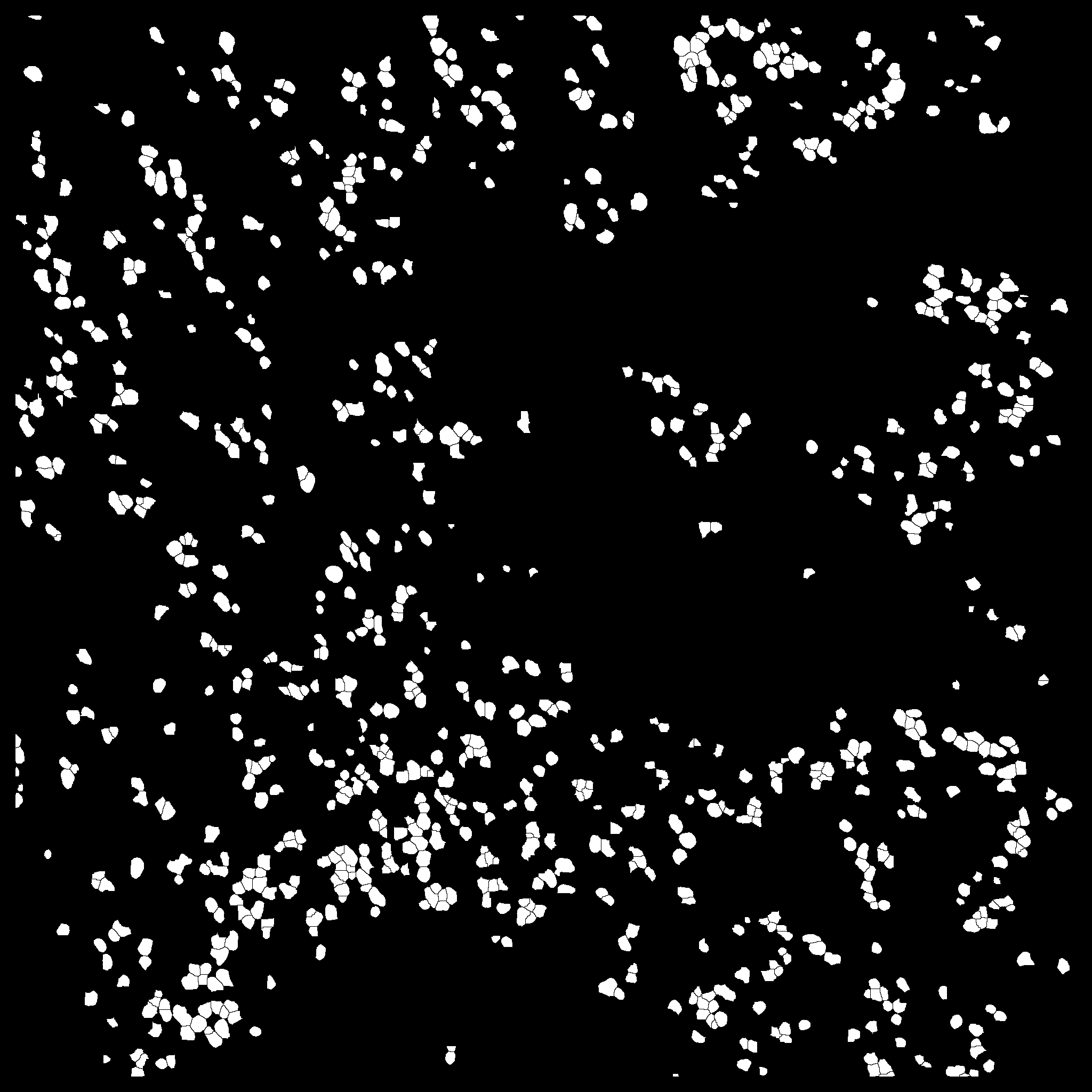

Supplement: Supplementary file 6 — Source Data [file 41467_2023_42878_MOESM6_ESM.zip › FigS9-S10/Patient37/otherRefCellsMask.png]

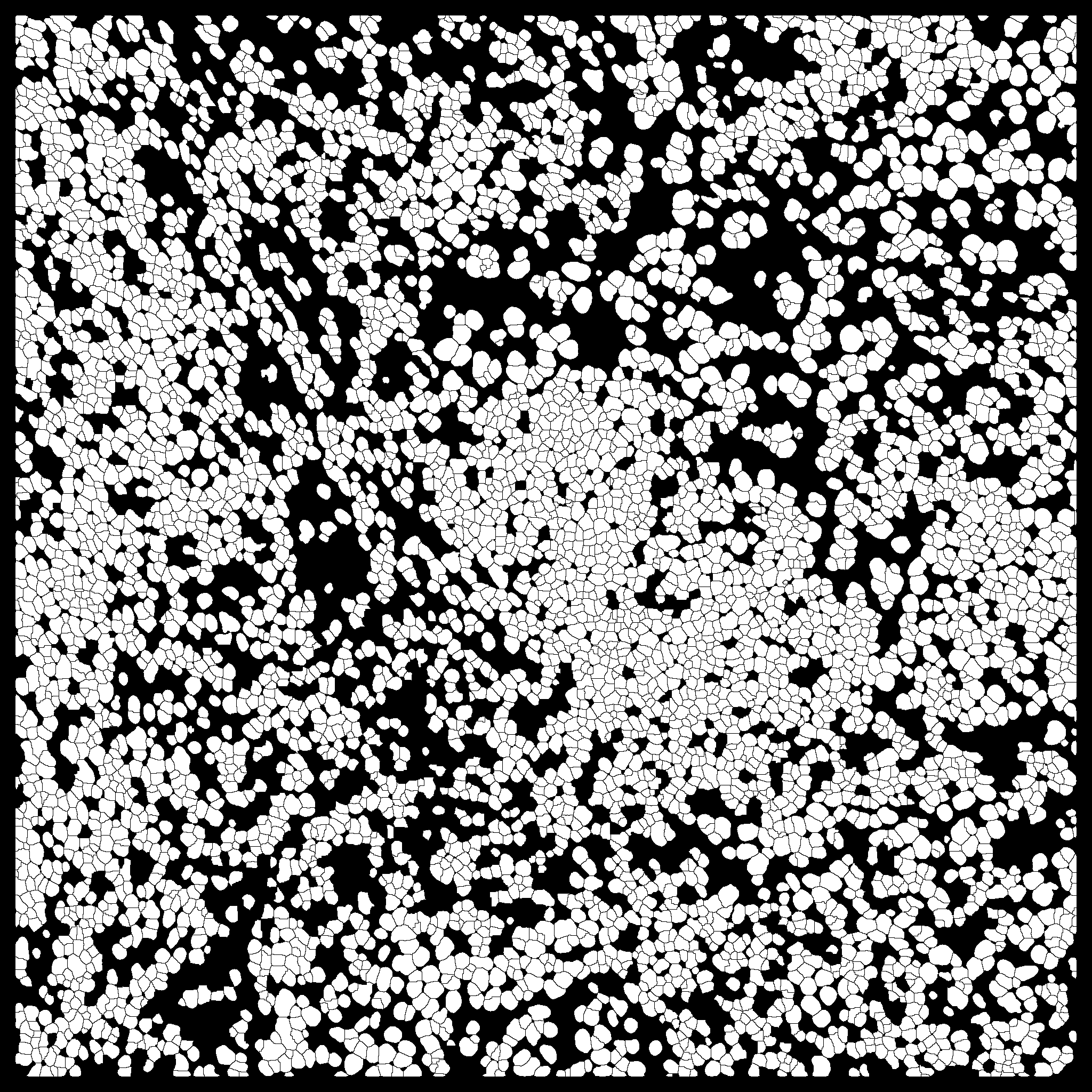

Supplement: Supplementary file 6 — Source Data [file 41467_2023_42878_MOESM6_ESM.zip › FigS9-S10/Patient37/othersMask.png]

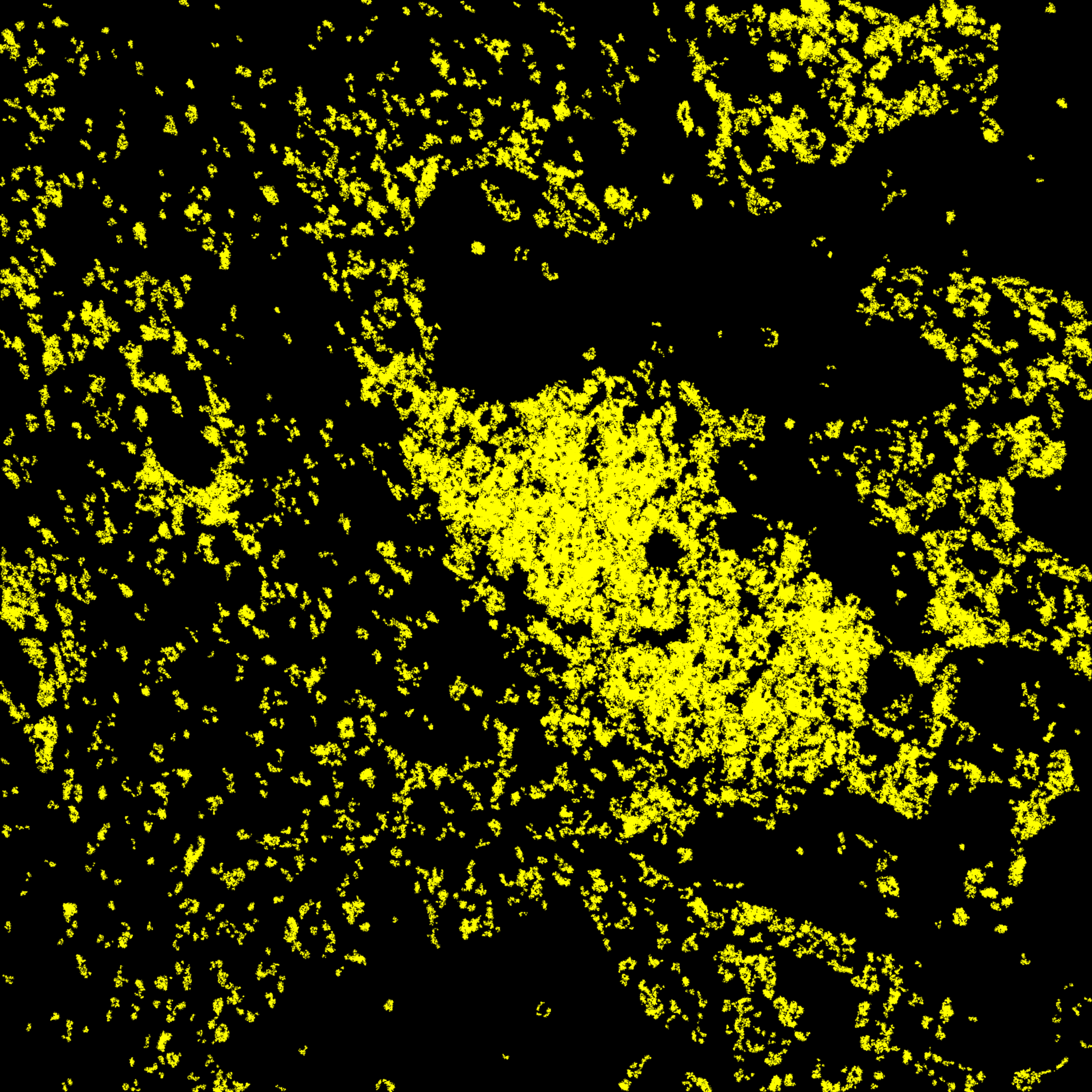

Supplement: Supplementary file 6 — Source Data [file 41467_2023_42878_MOESM6_ESM.zip › FigS9-S10/Patient37/TA459_multipleCores2_Run-4_Point37_Overlay.tiff]

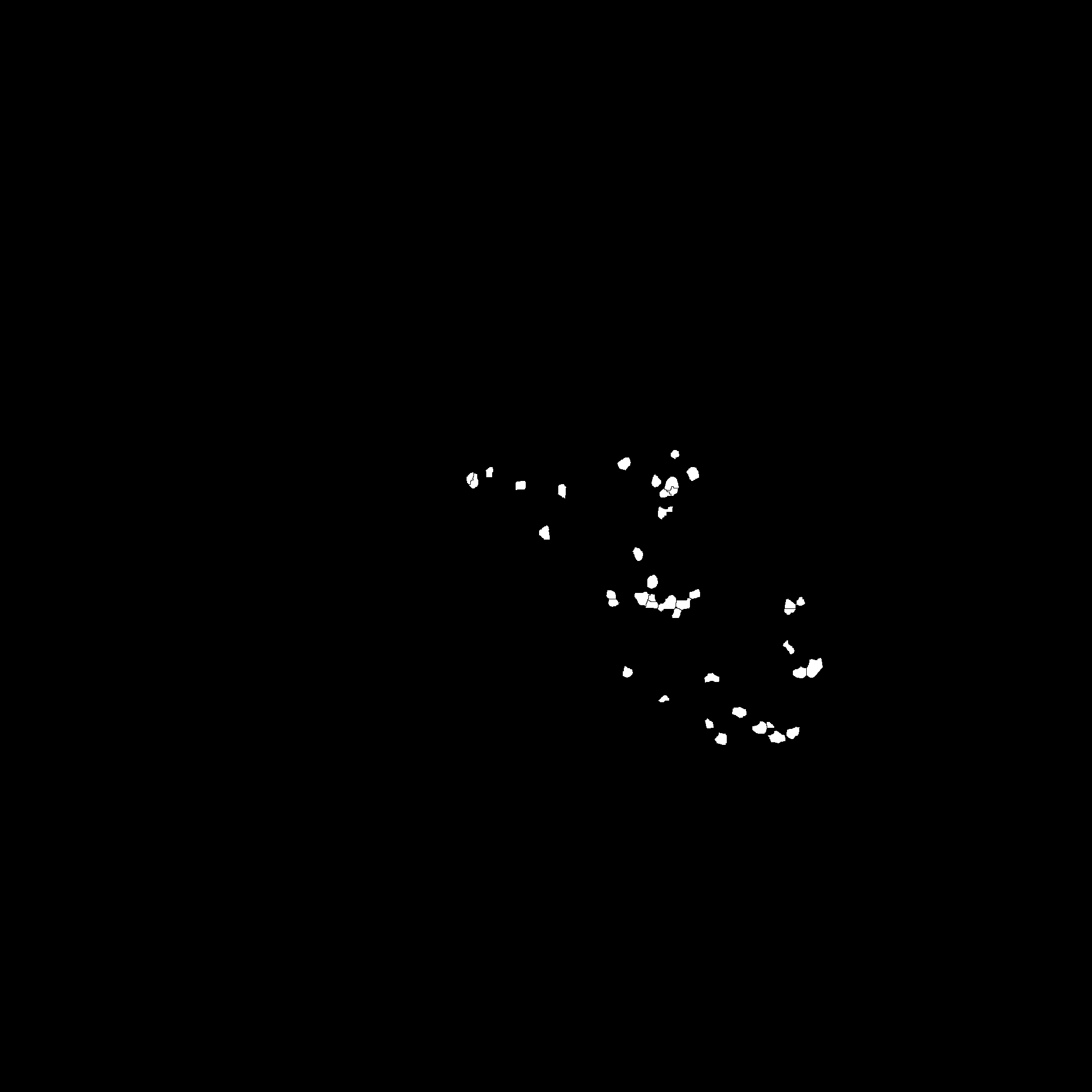

Supplement: Supplementary file 6 — Source Data [file 41467_2023_42878_MOESM6_ESM.zip › FigS9-S10/Patient37/refCellsInNicheMask.png]

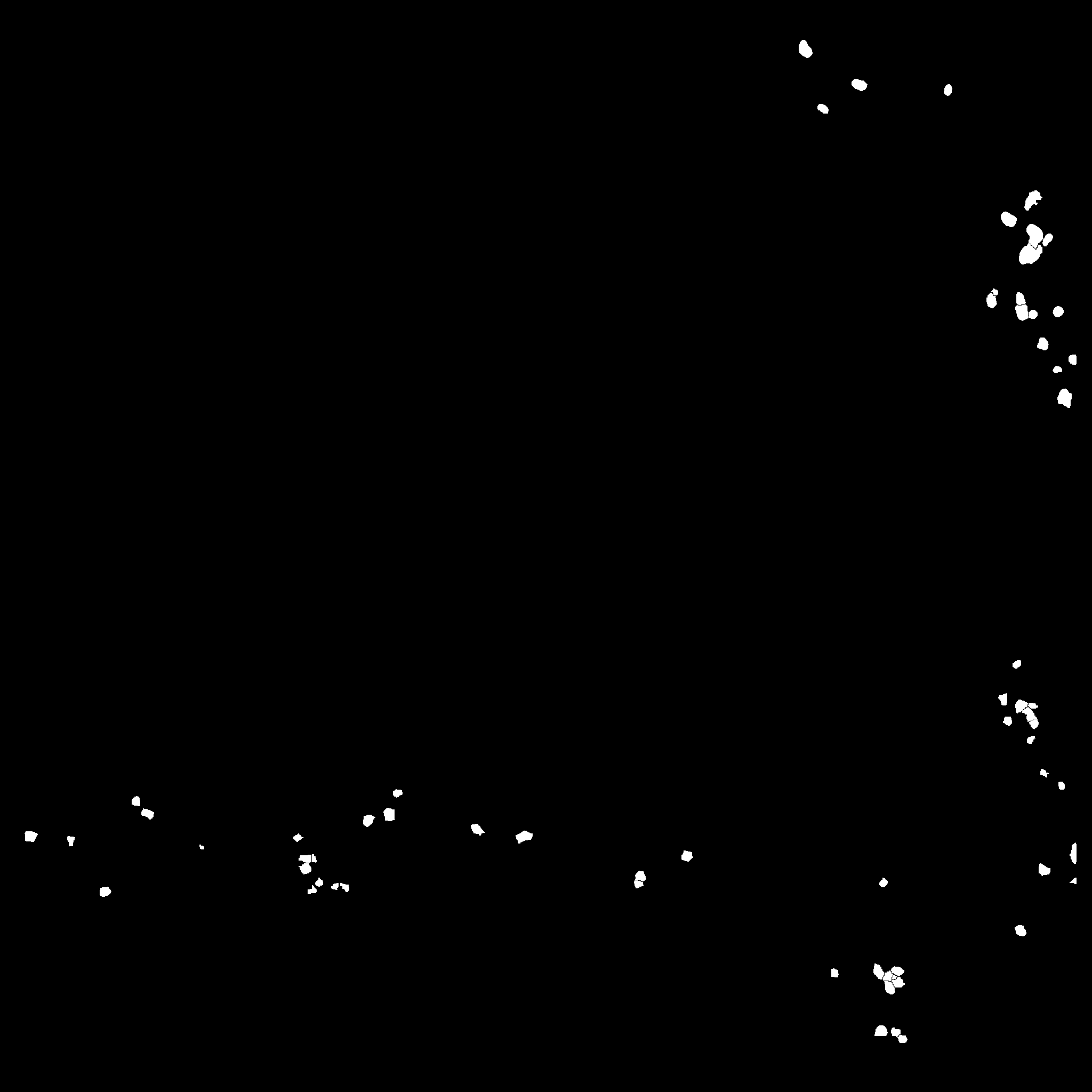

Supplement: Supplementary file 6 — Source Data [file 41467_2023_42878_MOESM6_ESM.zip › FigS9-S10/Patient28/otherRefCellsMask.png]

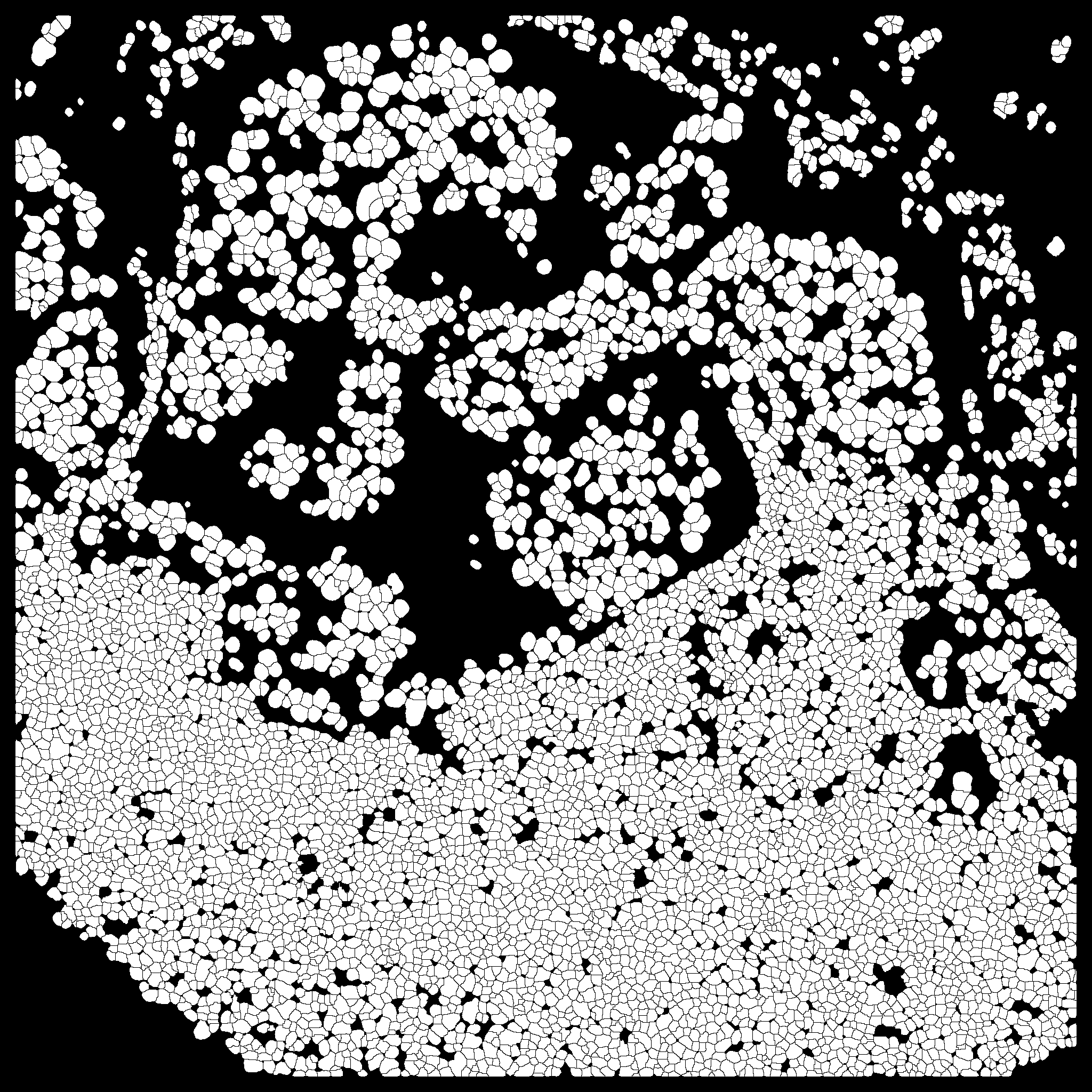

Supplement: Supplementary file 6 — Source Data [file 41467_2023_42878_MOESM6_ESM.zip › FigS9-S10/Patient28/othersMask.png]

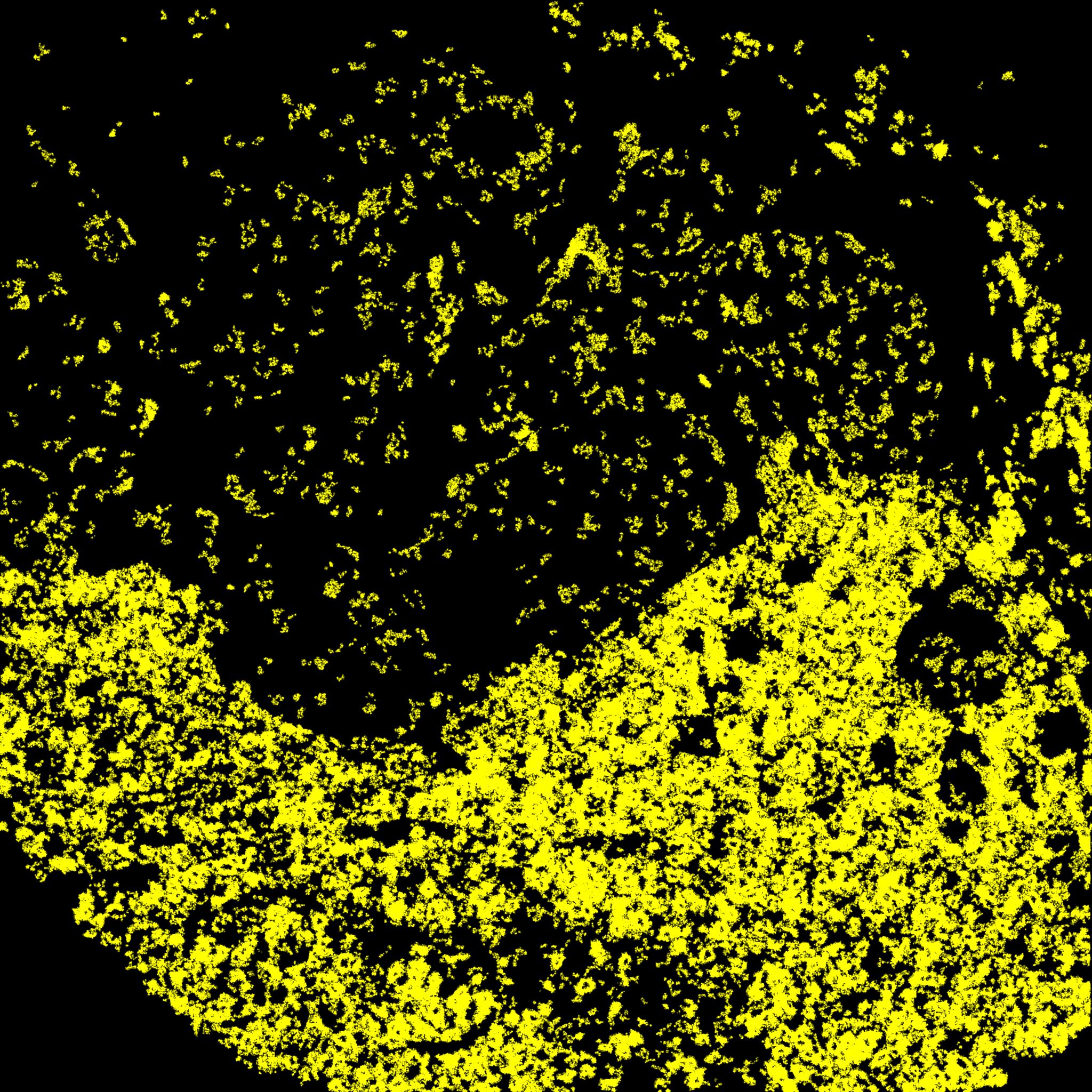

Supplement: Supplementary file 6 — Source Data [file 41467_2023_42878_MOESM6_ESM.zip › FigS9-S10/Patient28/TA459_multipleCores2_Run-4_Point28_Overlay.tiff]

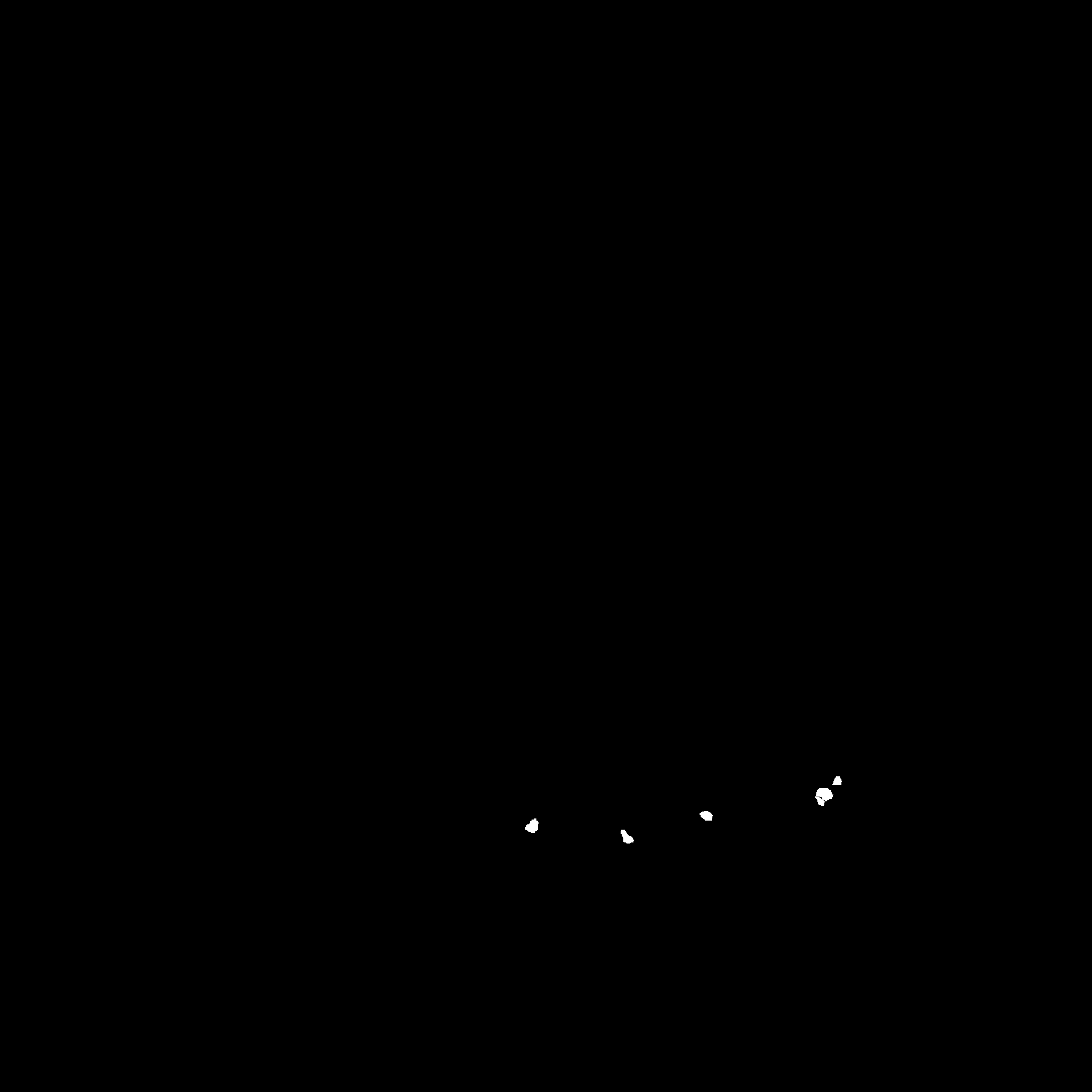

Supplement: Supplementary file 6 — Source Data [file 41467_2023_42878_MOESM6_ESM.zip › FigS9-S10/Patient28/refCellsInNicheMask.png]

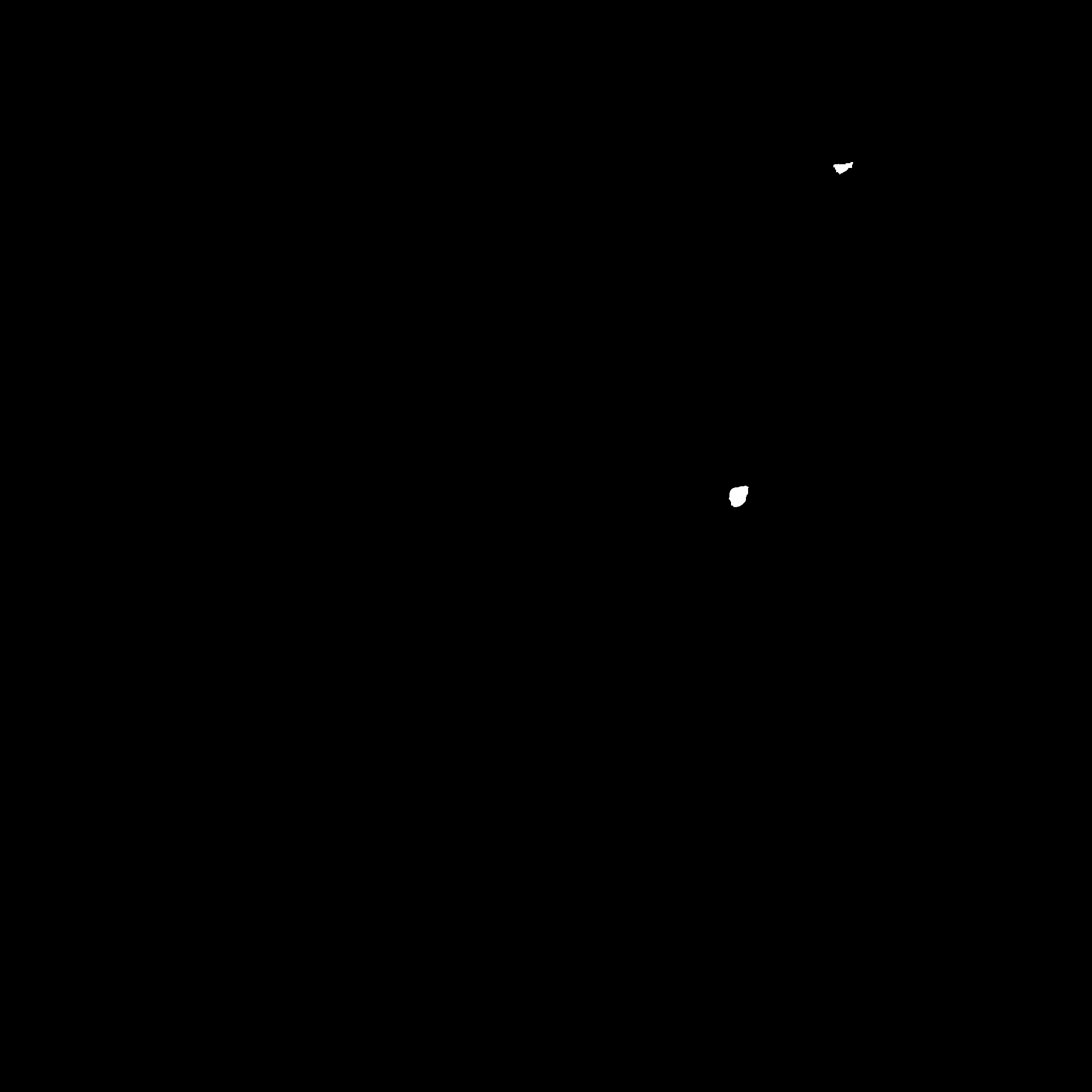

Supplement: Supplementary file 6 — Source Data [file 41467_2023_42878_MOESM6_ESM.zip › FigS9-S10/Patient19/otherRefCellsMask.png]

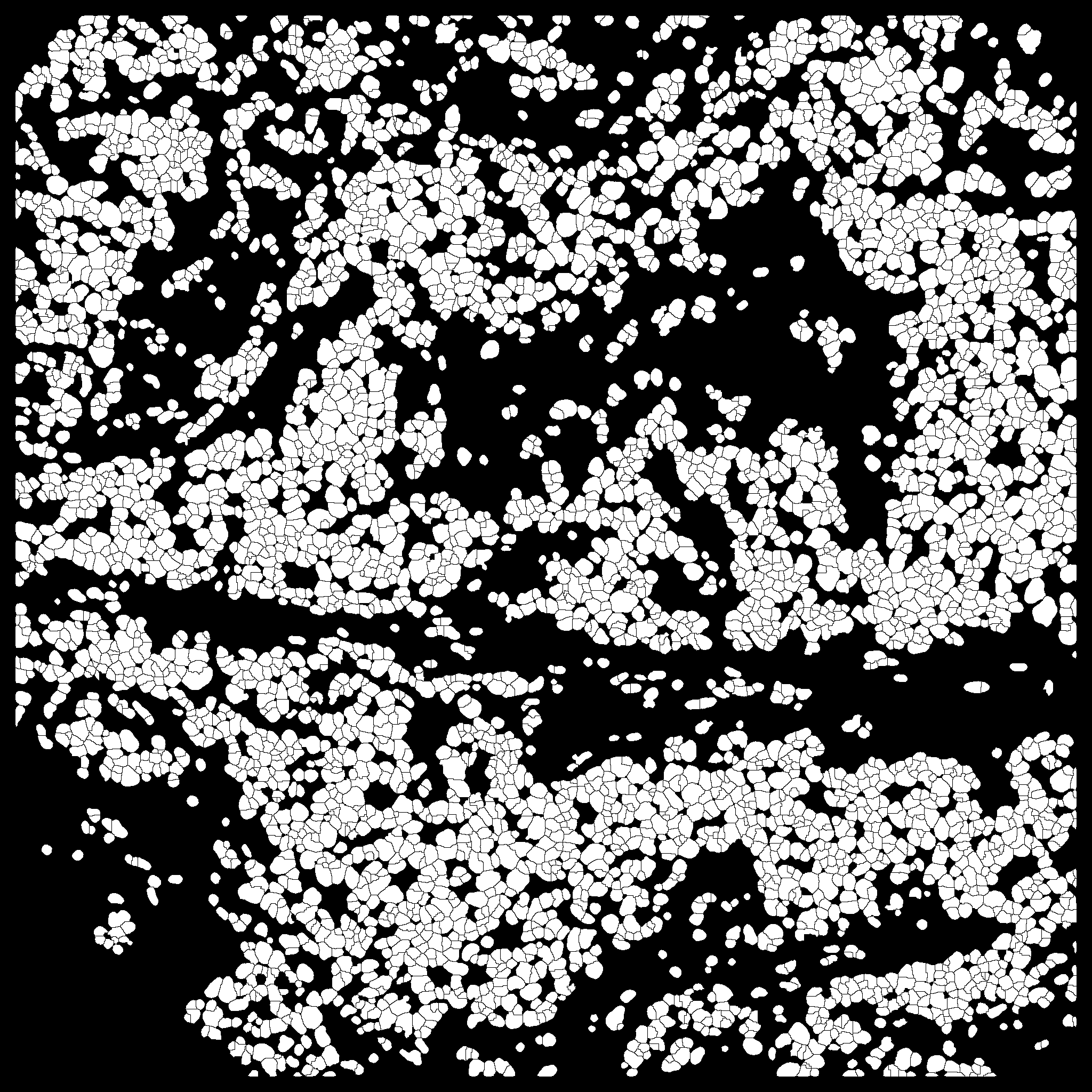

Supplement: Supplementary file 6 — Source Data [file 41467_2023_42878_MOESM6_ESM.zip › FigS9-S10/Patient19/othersMask.png]

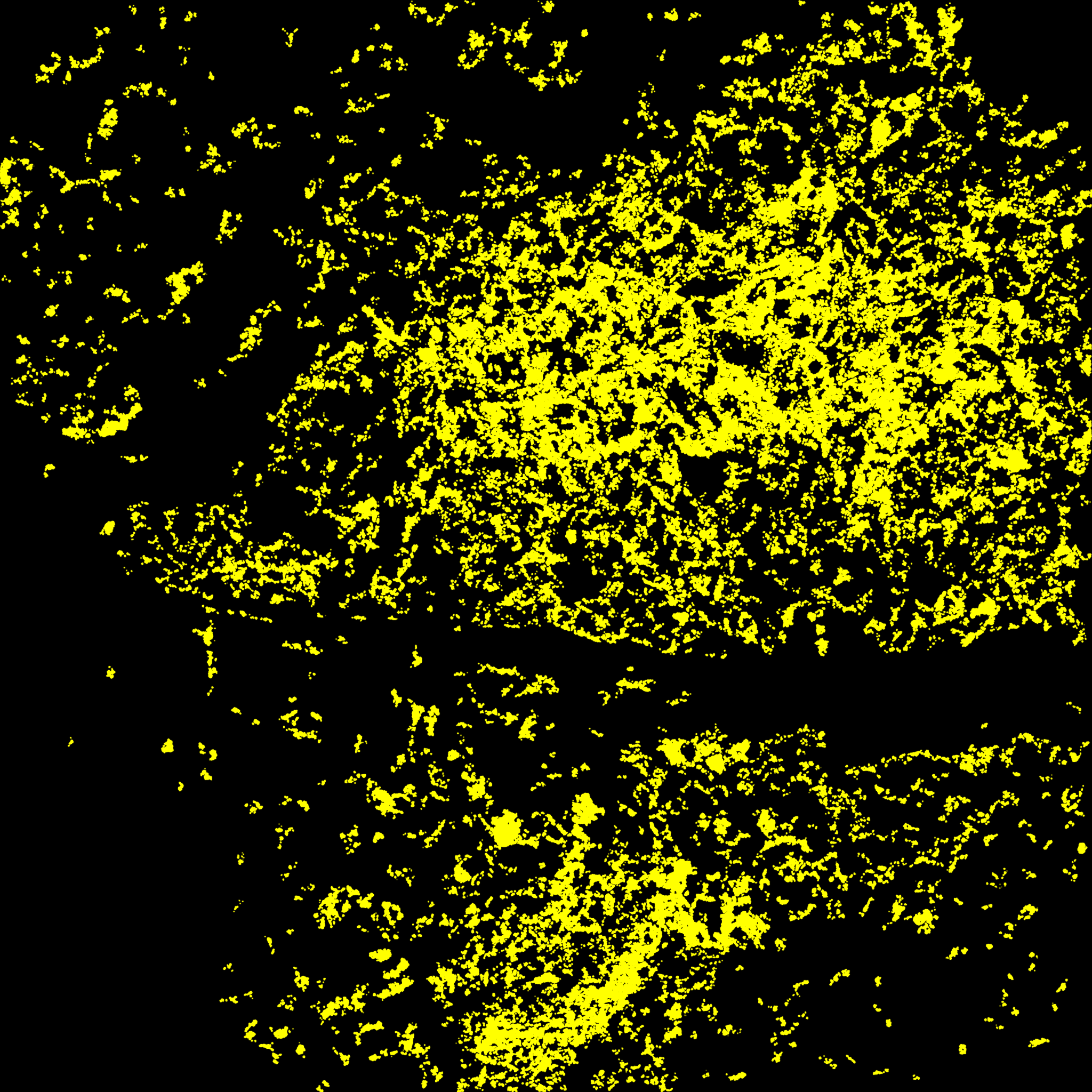

Supplement: Supplementary file 6 — Source Data [file 41467_2023_42878_MOESM6_ESM.zip › FigS9-S10/Patient19/TA459_multipleCores2_Run-4_Point19_Overlay.tiff]

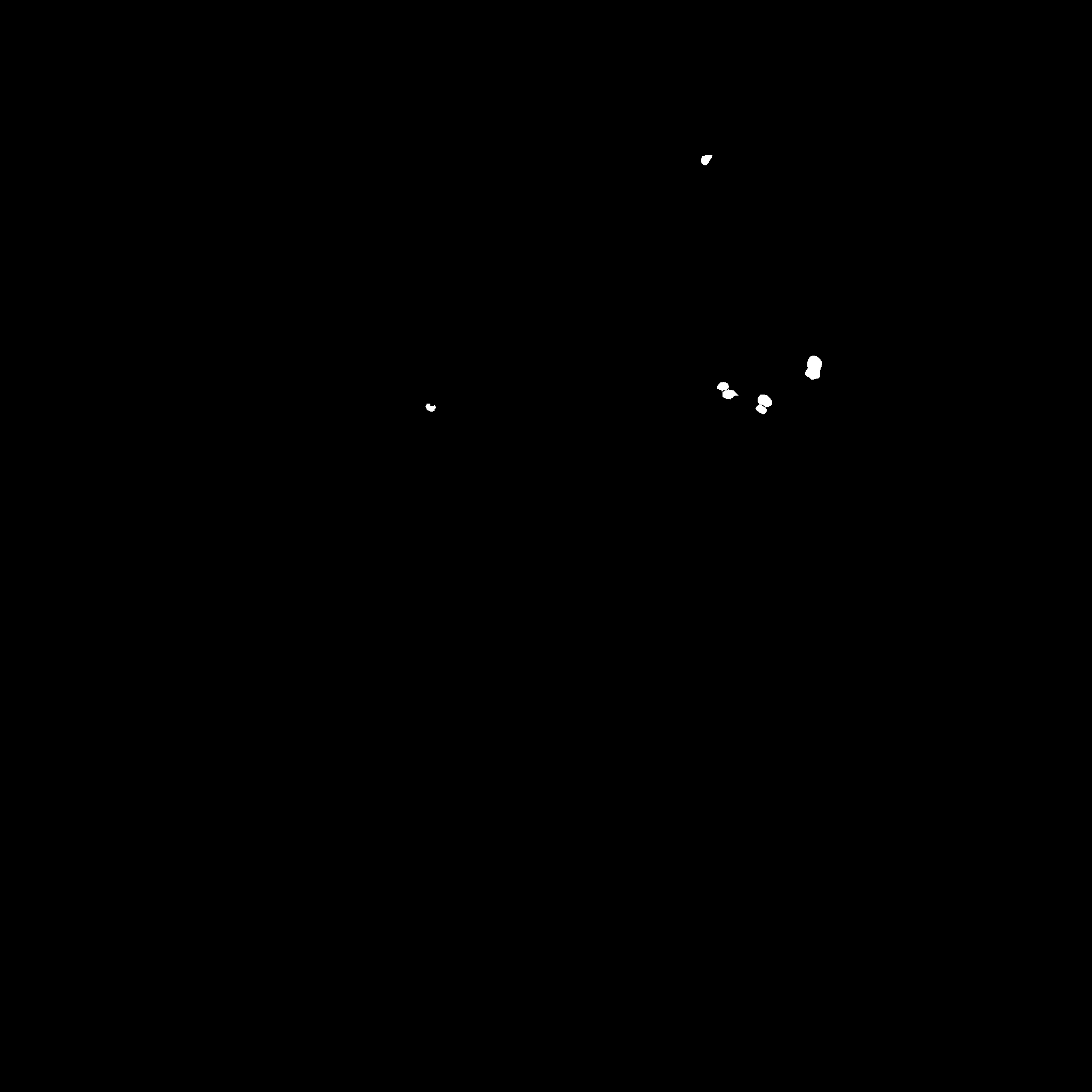

Supplement: Supplementary file 6 — Source Data [file 41467_2023_42878_MOESM6_ESM.zip › FigS9-S10/Patient19/refCellsInNicheMask.png]

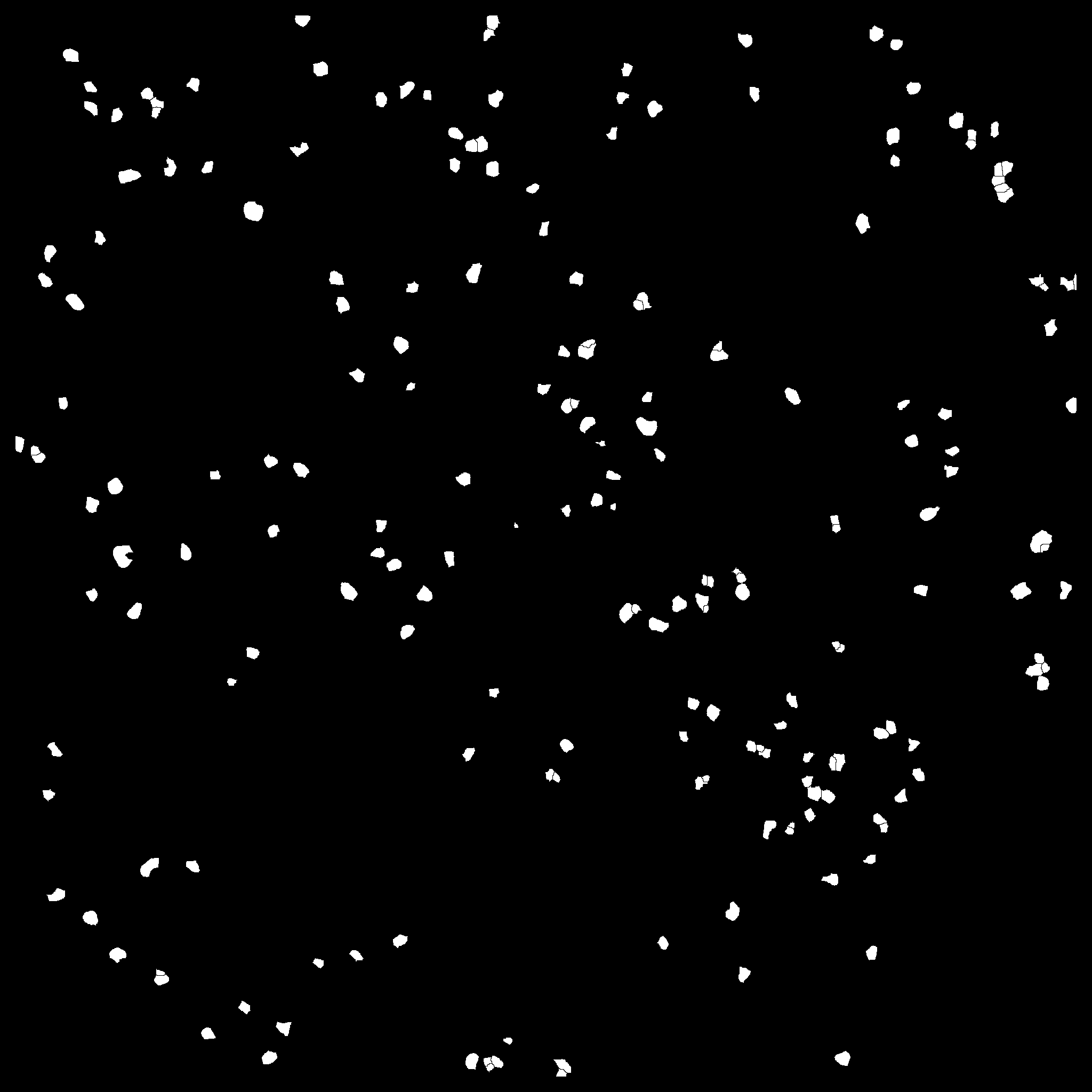

Supplement: Supplementary file 6 — Source Data [file 41467_2023_42878_MOESM6_ESM.zip › FigS9-S10/Patient12/otherRefCellsMask.png]

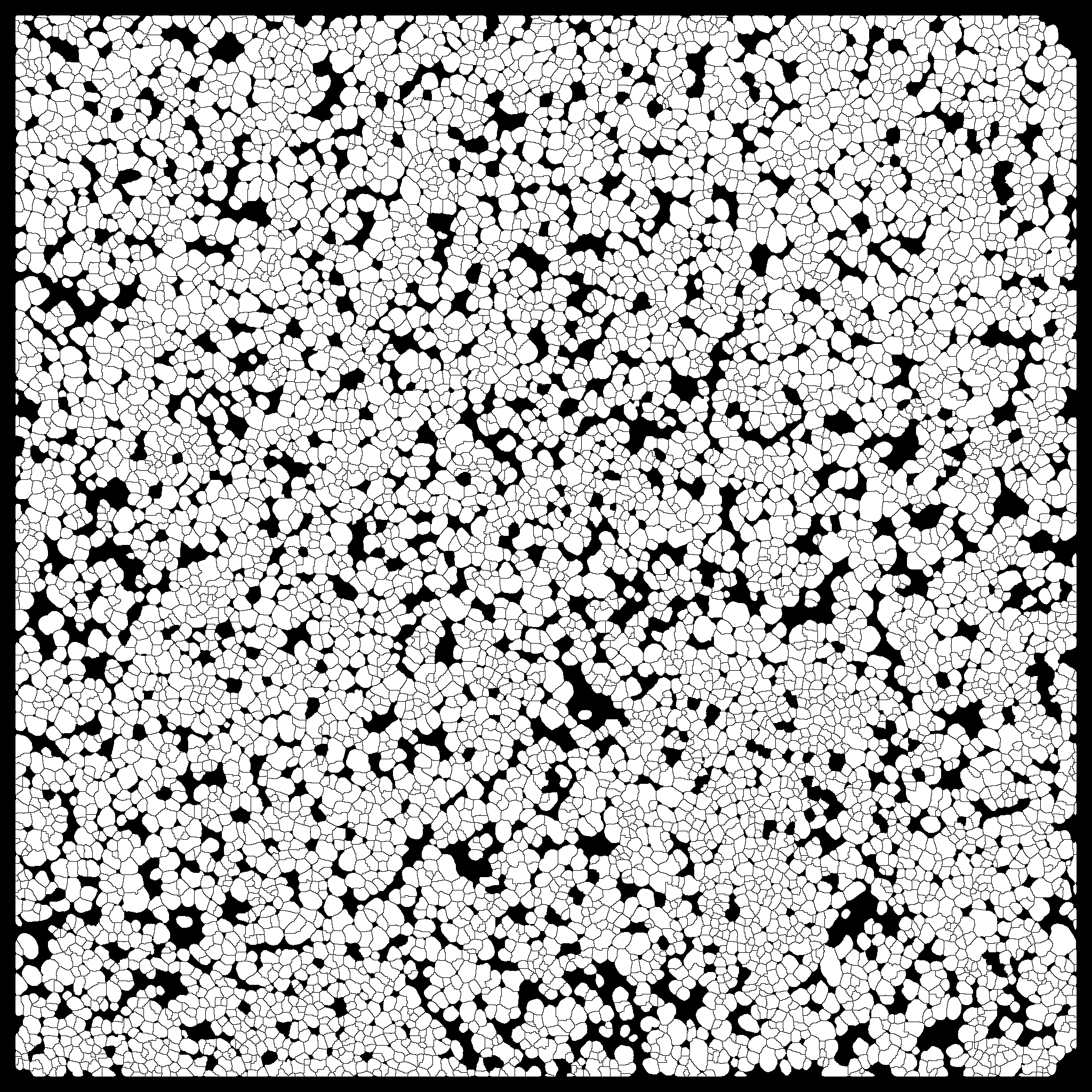

Supplement: Supplementary file 6 — Source Data [file 41467_2023_42878_MOESM6_ESM.zip › FigS9-S10/Patient12/othersMask.png]

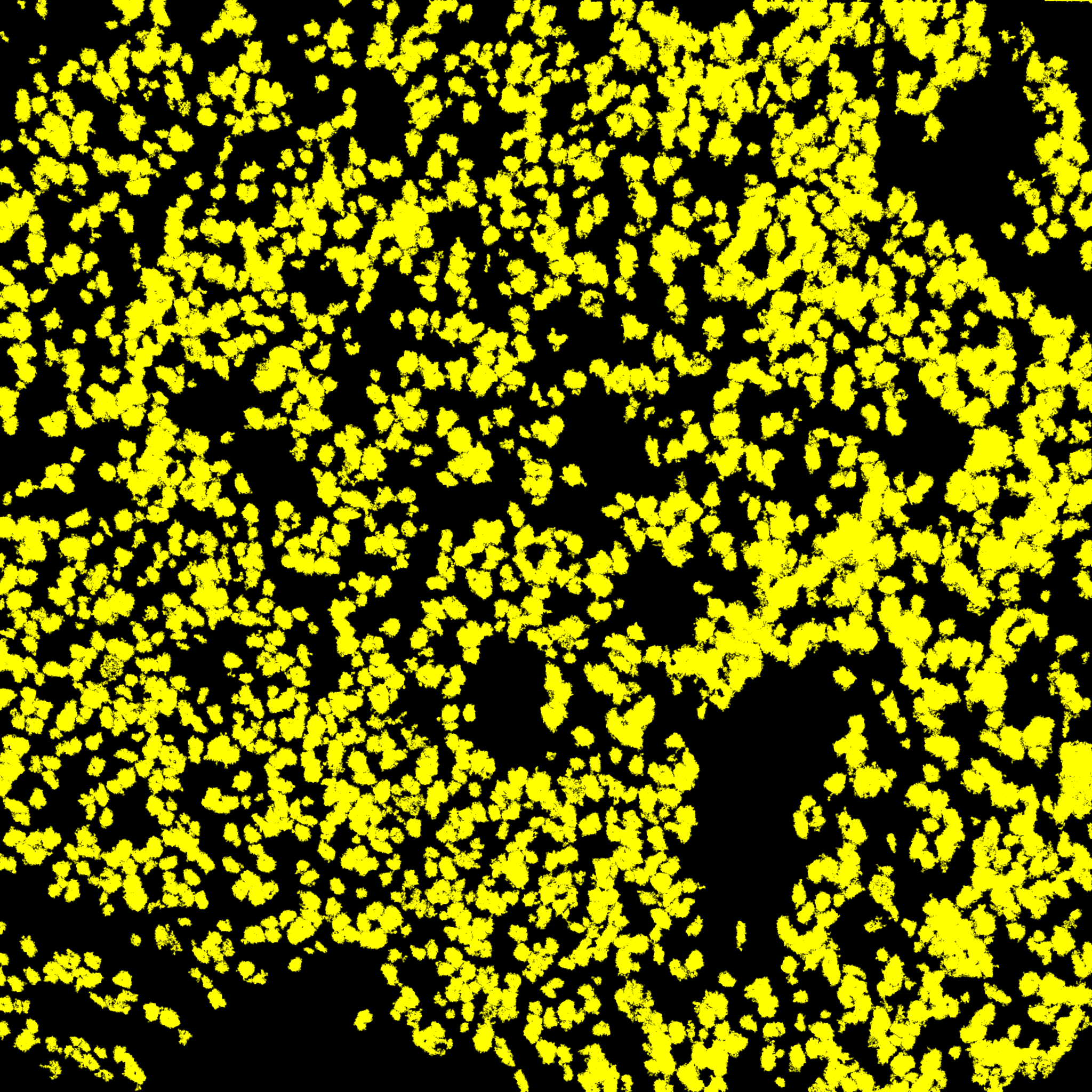

Supplement: Supplementary file 6 — Source Data [file 41467_2023_42878_MOESM6_ESM.zip › FigS9-S10/Patient12/TA459_multipleCores2_Run-4_Point12_Overlay.tiff]

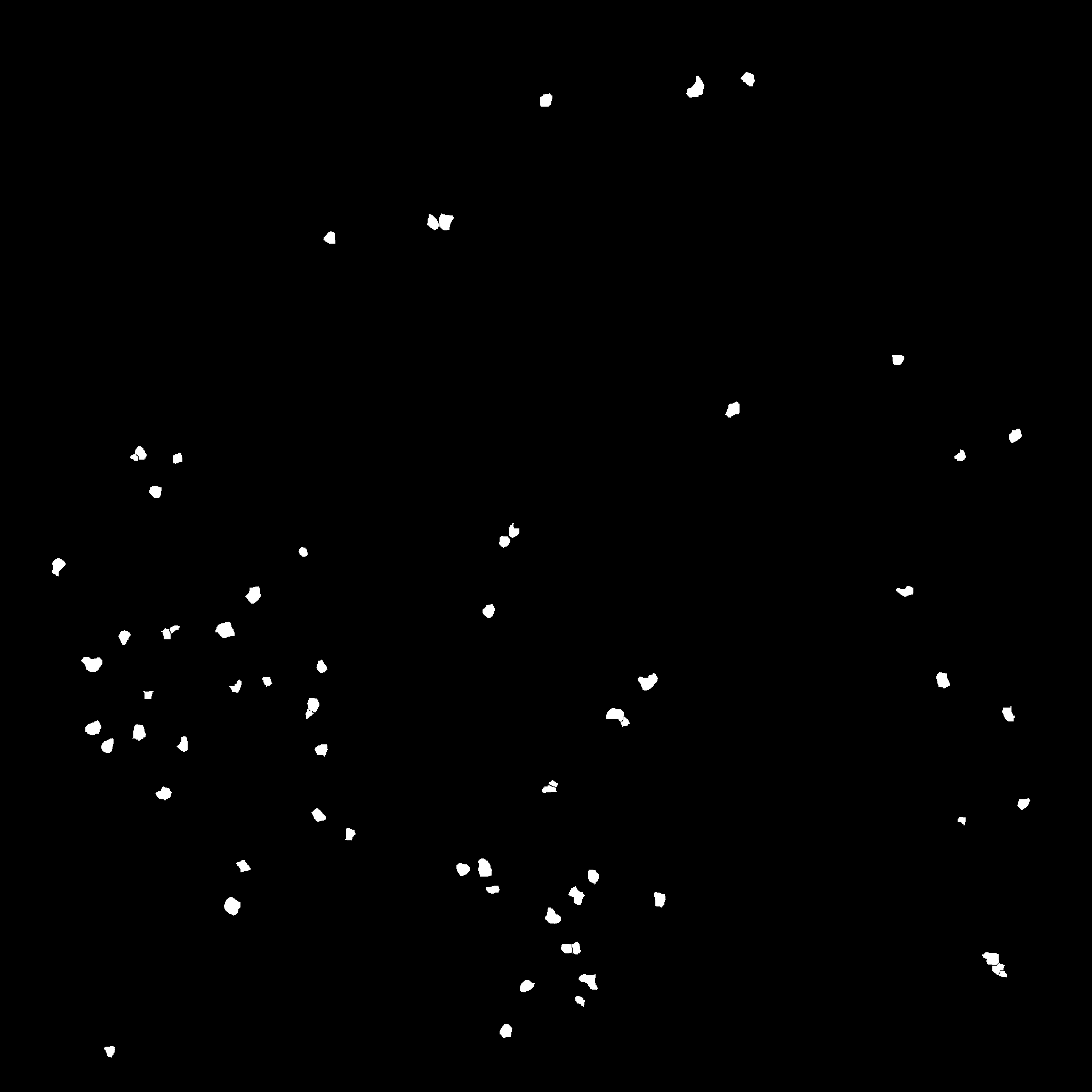

Supplement: Supplementary file 6 — Source Data [file 41467_2023_42878_MOESM6_ESM.zip › FigS9-S10/Patient12/refCellsInNicheMask.png]

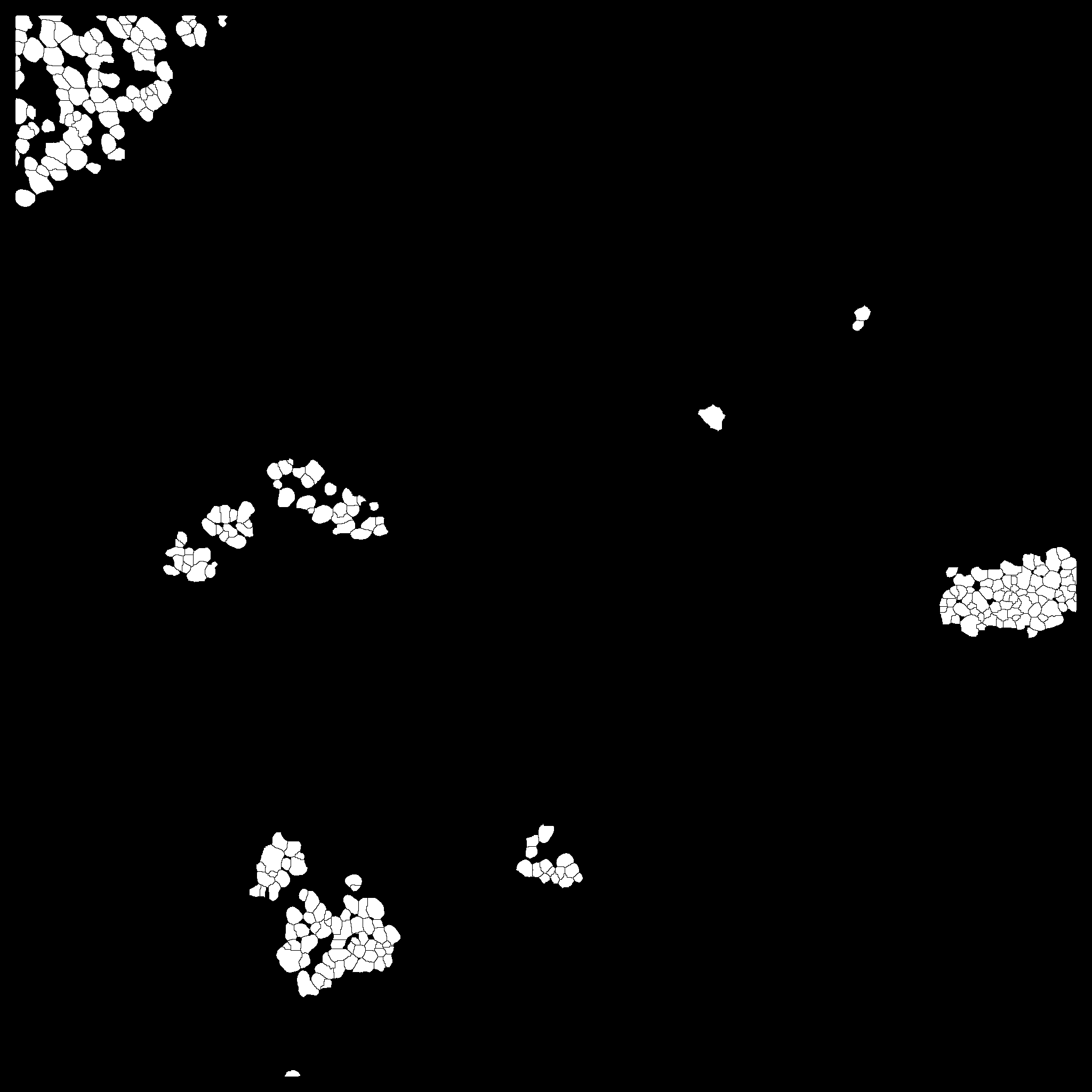

Supplement: Supplementary file 6 — Source Data [file 41467_2023_42878_MOESM6_ESM.zip › FigS9-S10/Patient35/otherRefCellsMask.png]

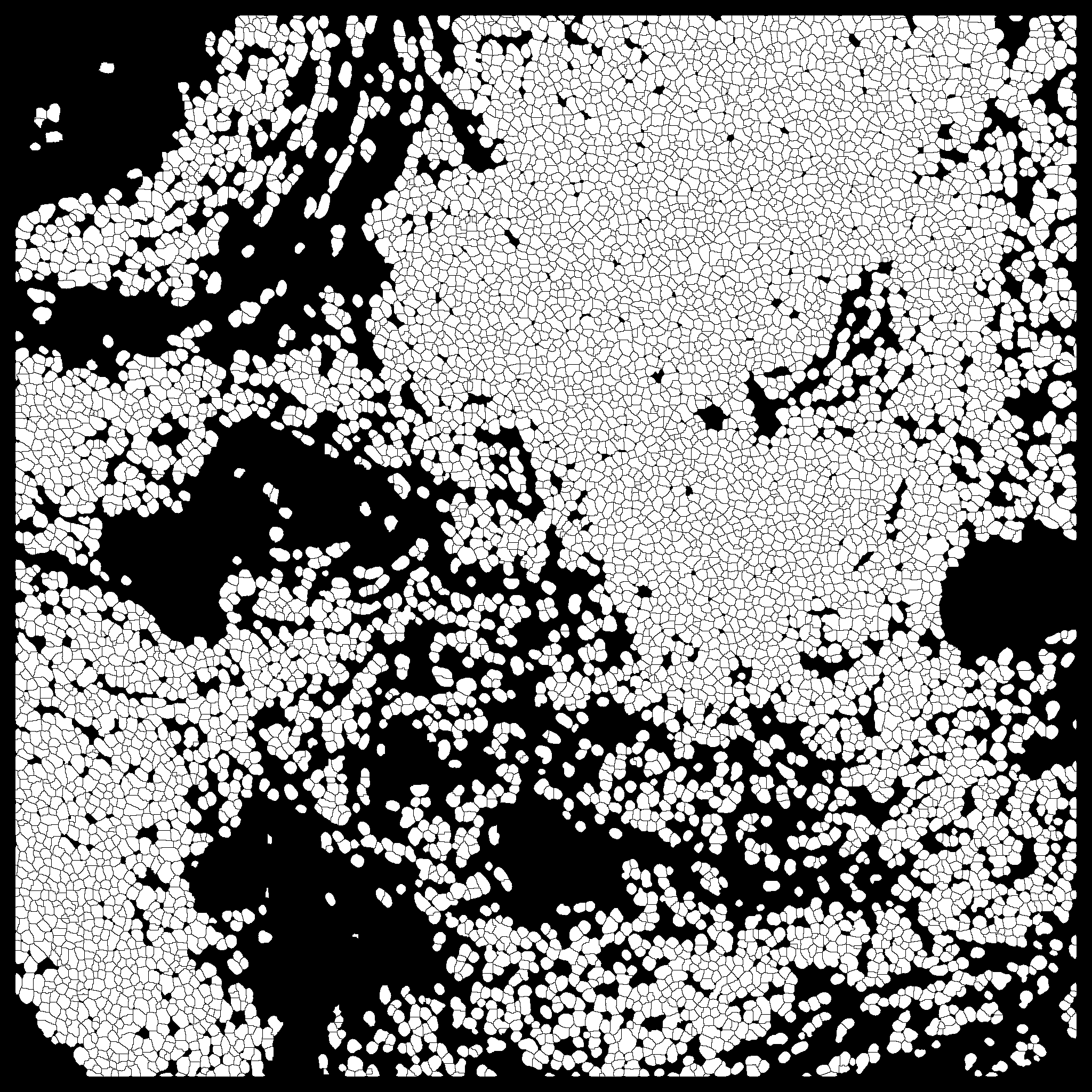

Supplement: Supplementary file 6 — Source Data [file 41467_2023_42878_MOESM6_ESM.zip › FigS9-S10/Patient35/othersMask.png]

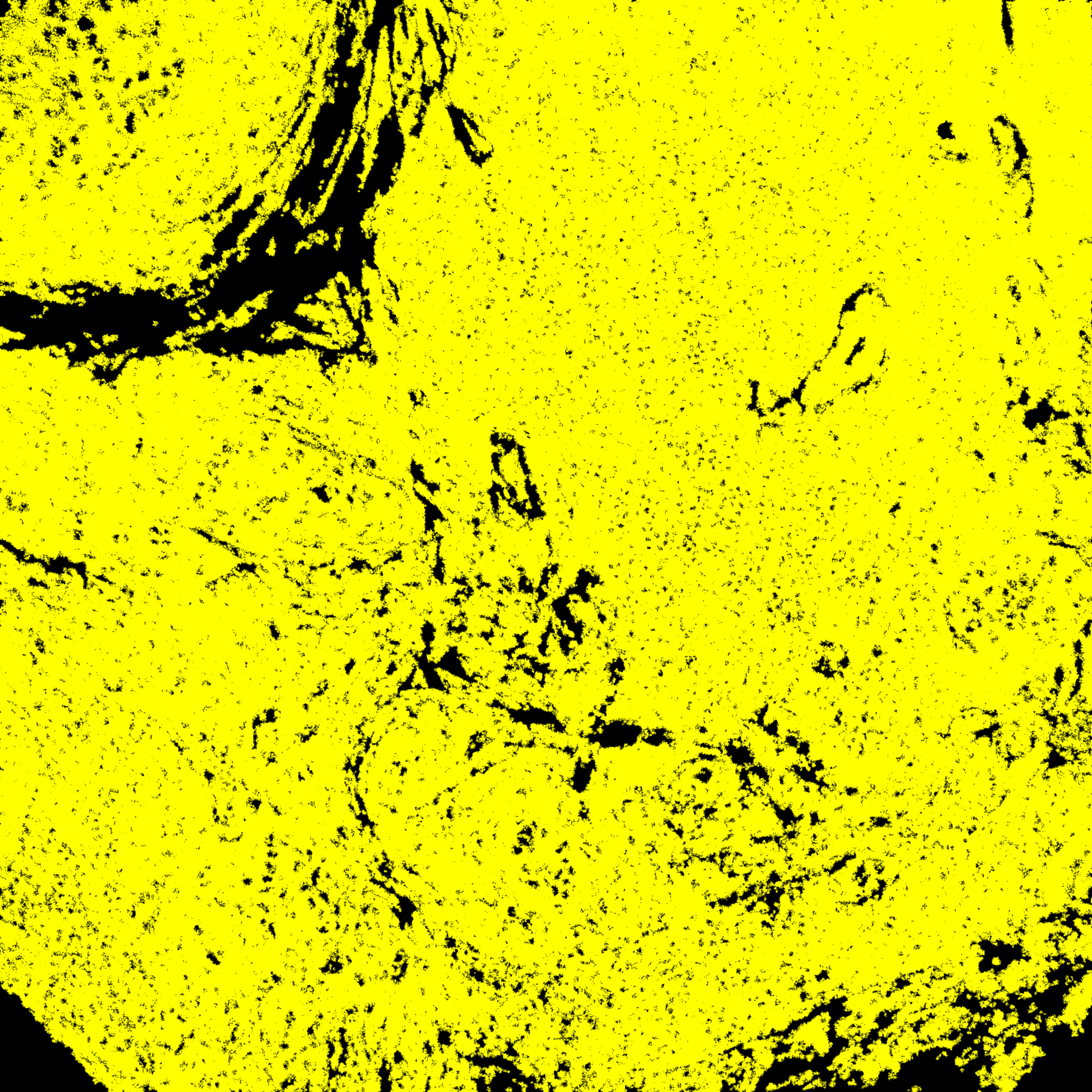

Supplement: Supplementary file 6 — Source Data [file 41467_2023_42878_MOESM6_ESM.zip › FigS9-S10/Patient35/TA459_multipleCores2_Run-4_Point35_Overlay.tiff]

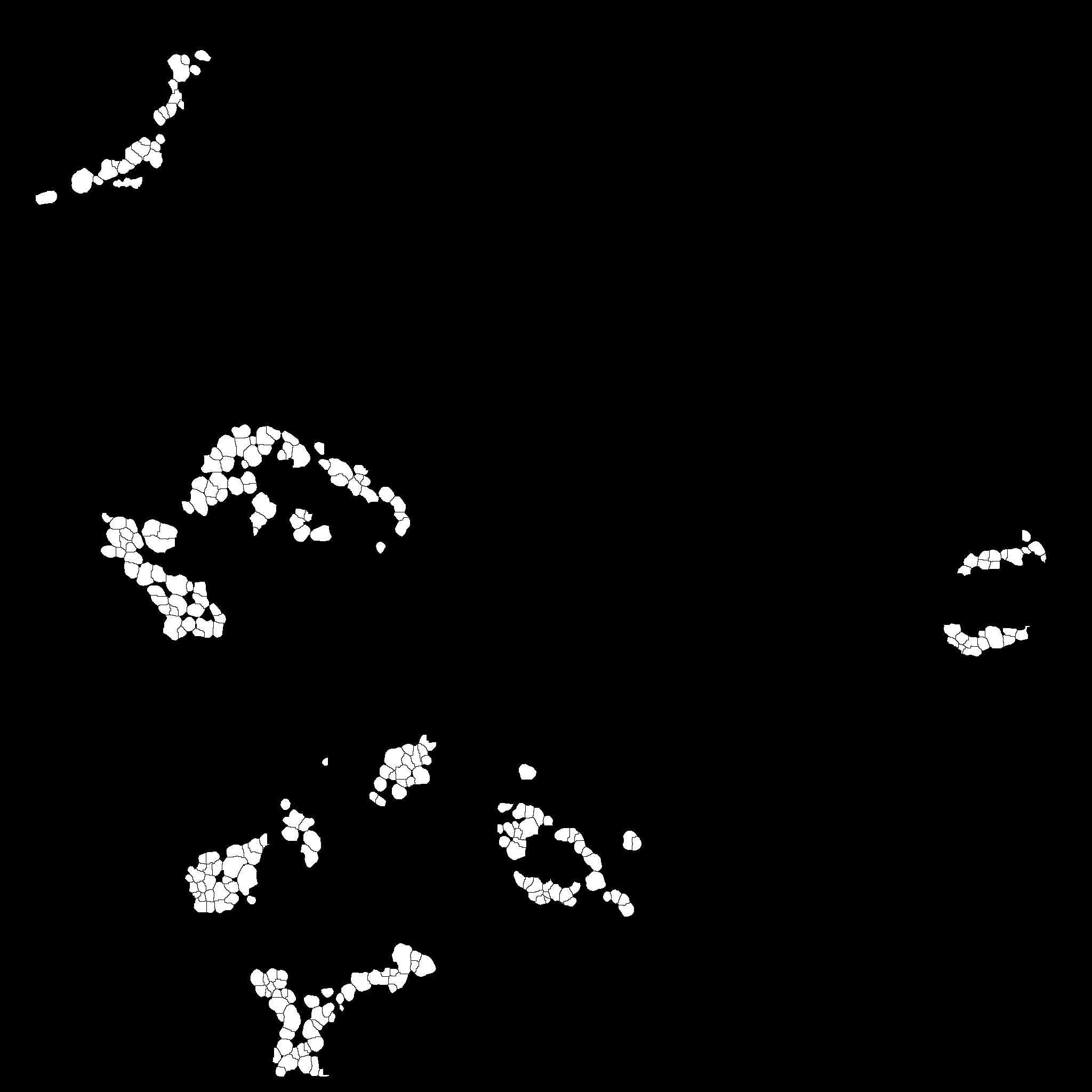

Supplement: Supplementary file 6 — Source Data [file 41467_2023_42878_MOESM6_ESM.zip › FigS9-S10/Patient35/refCellsInNicheMask.png]
